# Ultrasensitive Circularly Polarized Photon Detectors Based on Chiral Two-Dimensional MoS_2_

**DOI:** 10.1021/acsnano.5c12182

**Published:** 2025-10-23

**Authors:** Ye Wang, Yiru Zhu, Tieyuan Bian, Ziwei Jeffery Yang, Yuanyuan Zhao, Han Yan, Yang Li, Yan Wang, Feng Ding, Jun Yin, Manish Chhowalla

**Affiliations:** † Department of Materials Science & Metallurgy, 2152 University of Cambridge; 27 Charles Babbage Road, Cambridge CB3 0FS, United Kingdom; ‡ Department of Applied Physics, 26680 The Hong Kong Polytechnic University; Kowloon, Hong Kong 999077, P.R.China; § Institute of Technology for Carbon Neutrality, Shenzhen Institute of Advanced Technology, Chinese Academy of Sciences, Shenzhen 518055, P.R.China; ∥ Suzhou Laboratory, Suzhou 215123, P.R.China

**Keywords:** Two-dimensional materials, transition metal dichalcogenides, chirality, circularly polarized light, circular dichroism

## Abstract

Engineering chiral optical and electronic properties of materials is interesting for applications in sensing and quantum information. State-of-the-art chiral optoelectronic devices are mostly based on three-dimensional (3D) and quasi-two-dimensional (2D) materials. Here we demonstrate chiral 2D MoS_2_ with sub-nanometer thickness via chirality transfer from l-/d-penicillamine (l-/d-PEN). We report a giant molar ellipticity of 10^8^ deg·cm^2^/dmol in monolayer solid-state films, up to 3 orders of magnitude higher than 3D chiral materials. Phototransistors with chiral 2D MoS_2_ channels exhibit gate-tunable circularly polarized light detection with responsivity of >10^2^ A/W and anisotropy *g*-factor of 1.98, close to the theoretical maximum of 2.0. The reduced dimensionality magnifies the chirality transfer efficiency, allowing realization of ultrasensitive detectors for circularly polarized photons.

## Graphical abstract

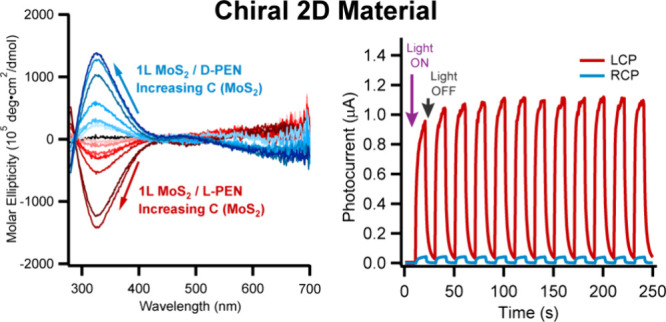

## Introduction

Chirality describes the absence of mirror symmetry.^1,2^ Chiral materials and structures can be used as sensors for stereoselective recognition^3^ and chemical processes for enantioselective catalysis.^4^ Chiral states of photons or electrons can be used for information transmission in quantum networks.^5–7^ Solid-state chiral materials with technologically interesting optical and electrical properties have just begun to emerge. Recent work has shown that it is possible to transfer chirality from chiral organic molecules onto inorganic materials.^8–11^ This provides a pathway for realizing chiral materials that are optically and electronically active so that high-performance devices such as photoemitters^12–15^ and photodetectors^16–19^ for emission and detection of circularly polarized light can be realized.

Monolayer transition metal dichalcogenides (TMDs) possess excellent optical and electronic properties such as strong photoluminescence, single photon emission, high field effect mobility, high on/off ratio, and low subthreshold swing.^20–29^ In this work, we demonstrate that chirality transfer can be realized in sub-nanometer-thick semiconductors without deterioration of their optical and electronic properties. It is well-known that the symmetry of geometrically and optically achiral materials can be tuned to be chiral by surface functionalization.^8,10,17,19,30–35^ In multilayer or bulk MoS_2_, chirality induction in solution has been demonstrated via molecular functionalization primarily at the edges that leads to preferential folding of the MoS_2_ sheets, making it challenging to realize practical devices.^36^ Therefore, chirality induction in flat monolayer MoS_2_ is preferred. Here we use natural chiral amino acids containing thiol groups to functionalize flat 2D TMDs [Figure 1a]. Specifically, we have selected l-/d-penicillamine (l-/d-PEN) as the chiral molecules for functionalization of monolayer MoS_2_. Hybridization between thiols and sulfur vacancies in monolayer MoS_2_ results in efficient chirality transfer and molar ellipticity that surpass other 3D and quasi-2D materials. The strong chiral light–matter interactions in functionalized monolayer MoS_2_ allow the realization of gate-tunable photocurrent with ultrahigh sensitivity at room temperature and in ambient conditions.

**Figure 1:**
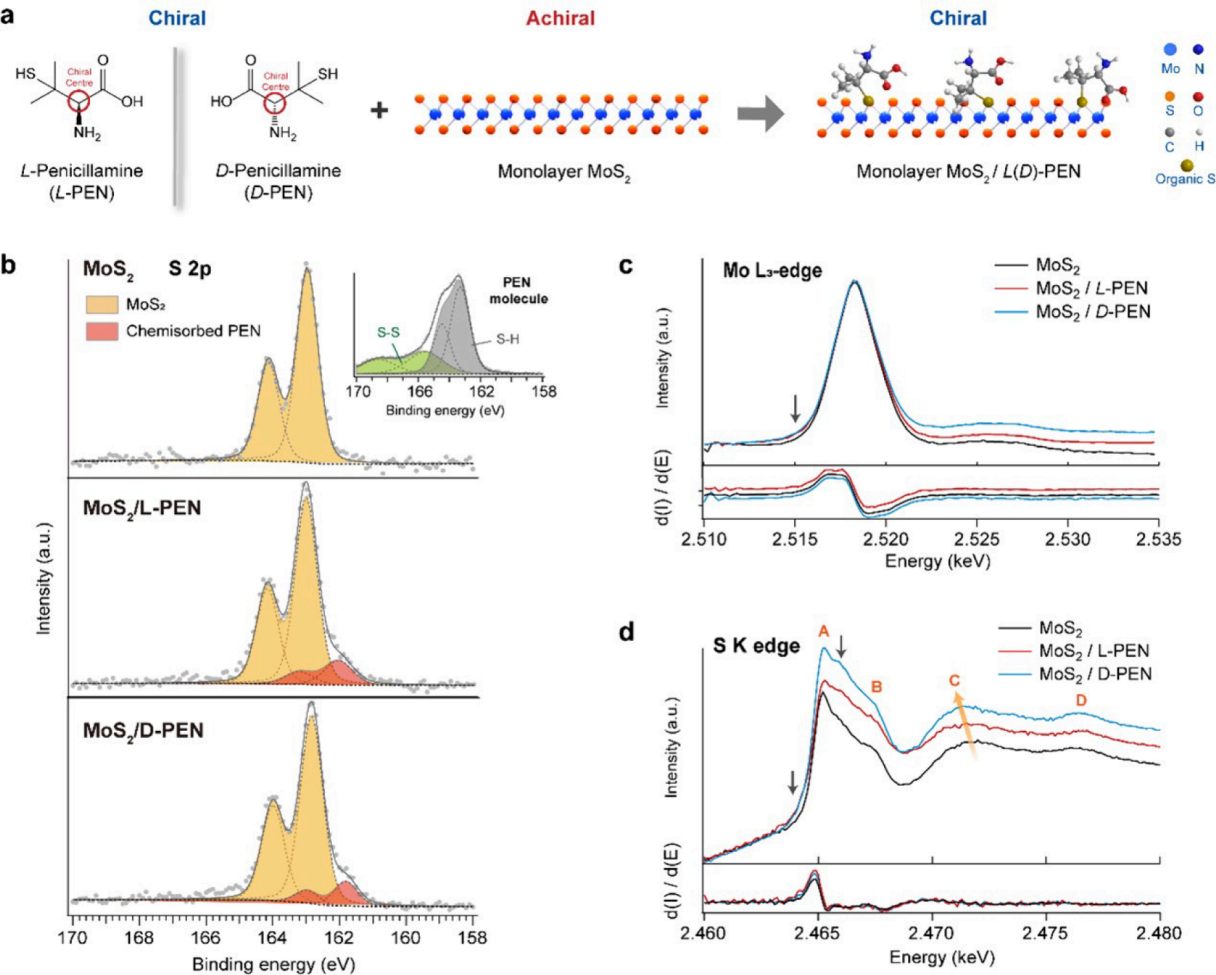
(a) Chemical structure of chiral molecules, l/d-Penicillamine (l/d-PEN) and schematic of monolayer MoS_2_. The schematic represents the general scheme for surface functionalization with chiral thiol molecules. Sulfur (marked as yellow balls) from thiol groups in PEN molecules is primarily attached at sulfur vacancies of MoS_2_ resulting in chemisorbed functionalization. (b) Core-level XPS S 2p spectra of MoS_2_, MoS_2_/l-PEN, and MoS_2_/d-PEN. In MoS_2_/l-PEN and MoS_2_/d-PEN the spectra show an additional component at lower binding energy (marked in red) than pure MoS_2_. The inset shows the S 2p spectrum of pure l-PEN molecules. (c, d) (c) Mo L_3_-edge and (d) S K-edge spectra of MoS_2_, MoS_2_/l-PEN, and MoS_2_/d-PEN. In panel c, the absorption edge is shown on top and its derivative d­(*I*)/d­(*E*) is shown below. The downward arrow at the edge shows that the absorption of functionalized samples is higher, which suggests a change in the core electron bonding energy due to functionalization. In panel d, the XANES of S edge is shown with the small downward arrow indicating that functionalized samples absorb more. The d­(*I*)/d­(*E*) curves for the three samples do not show significant differences. A, B, C, and D represent absorption bands from sulfur. The orange arrow shows that the C band disperses to lower energy.

## Results and Discussion

### Hybridization between Chiral Molecules and 2D MoS_2_

Functionalization of MoS_2_ by l-/d-PEN was initially characterized by X-ray photoelectron spectroscopy (XPS). As shown in Figure 1b, the core-level S 2p doublet of MoS_2_ appears at 162.94/164.18 eV. After functionalization with l-PEN, we observed an additional doublet peak at lower binding energy (162.19/163.42 eV, marked in red color). This doublet is typical of bound thiol and is attributed to reduced sulfur in the thiol that is chemically bonded with metal atoms.^37–39^ We have not found evidence of free PEN molecules that possess S 2p peaks at 163.37/164.45 eV (inset of Figure 1b), indicating that weakly bound free thiol molecules are largely absent in our samples. The degree of chemisorbed functionalization estimated from XPS measurements is 14.47% for MoS_2_/l-PEN and 12.02% for MoS_2_/d-PEN. Additional XPS spectra of Mo 3d, N 1s, and C 1s, atomic force microscopy (AFM), and Fourier-transform infrared (FTIR) spectroscopy (Figures S1–S3 in the Supporting Information) suggest that while signature functional groups of the molecule such as (−NH_2_, −COOH) can be probed on MoS_2_/l­(d)-PEN, molecules do not form aggregates or islands on MoS_2_ as indicated by the negligible (<0.1 nm) increase in roughness measured by AFM. Moreover, FTIR spectra show the absence of thiol groups (−SH) at 2598 cm^–1^ in MoS_2_/PEN, indicating conjugation of thiol molecules on MoS_2_ (Figure S3 in the Supporting Information).^38^ To probe the subtle changes induced by functionalization, we used synchrotron-based X-ray absorption near-edge structure (XANES) to obtain atomic-orbital-specific information before and after molecular functionalization of monolayer MoS_2_. Figure 1c shows the XANES Mo L_3_-edge (2p_3/2_ → 4d) of MoS_2_.^40,41^ No obvious shift or appearance of a shoulder at higher energy that would indicate the presence of oxidized species such as MoO_2_ (2524.5 eV) and MoO_3_ (2528.6 eV) was observed postfunctionalization.^42,41^ The d­(*I*)/d­(*E*) in Figure 1c shows a maximum at 2516.7 eV corresponding to the Mo^4+^ absorption edge of both pristine and functionalized MoS_2_. However, increased absorption as indicated by the downward arrow in Figure 1c at energies below the white line in functionalized samples was observed, indicating that the Mo orbitals in MoS_2_ are altered by PEN functionalization.^43^

The sulfur K-edge (1s → 3p) spectra are more sensitive to changes in the chemical environment of MoS_2_. Figure 1d shows that monolayer MoS_2_ exhibits four absorption bands, labeled as A, B, C, and D with peak maxima at 2465.2, 2467.3, 2471.9, and 2476.5 eV, respectively. The absorption edge according to the first derivative in Figure 1d is defined at 2464.9 eV. Compared to pristine MoS_2_, l- and d-PEN functionalized MoS_2_ display three prominent features: (1) in MoS_2_/l­(d)-PEN, the intensity ratio between A and B bands decreases (more evident comparison can be found in the normalized spectra in Figure S4 in the Supporting Information); (2) band C red-shifts 0.7 eV (from 2472.0 to 2471.3 eV); (3) similar to Mo L_3_-edge spectra, a weak pre-edge absorption occurs from 2463.5 to 2464.5 eV after molecular functionalization. The A and B bands have been attributed to absorption due to excitation of sulfur 1s core electrons excitation to p_*x*,*y*_ and p_*z*_ states, respectively.^44^ Band C is a broad merged double band corresponding to transition from S 1s electrons to p_*z*_ (lower energy) and p_*x*,*y*_ (higher energy) like states in the continuum, originating from hybridization with S 3d orbitals.^40,45^ We can therefore translate observations of a decrease in intensity ratio between A and B bands and a red shift of 0.68 eV to more 1s to 3p_*z*_ transitions following molecular functionalization, which may be attributed to the availability of more S p_*z*_ states provided by thiol groups from PEN molecules on MoS_2_. In all samples, we did not find absorption bands from free PEN molecules at 2473.1 eV,^46^ indicating that the changes in XANES are from sulfur orbitals modified by the molecule in MoS_2_. The XANES results suggest that evaporation of chiral PEN molecules onto monolayer MoS_2_ leads to a subtle but measurable orbital hybridization between the two.

### Ultrahigh Molar Ellipticity and Mechanism of Chirality Transfer in 2D

Next, we assess the chiroptical properties of functionalized 2D MoS_2_ by measuring circular dichroism (CD) spectra at room temperature. We prepare single monolayered films (either spin-coated from solution or CVD-grown) on quartz (surface coverage of ∼80%). CD (Δ*A*) is the difference in absorbance of left circularly polarized light (LCP) (*A*_LCP_) and right circularly polarized light (RCP) (*A*_RCP_):

1 $$\Delta A=A_{\mathrm{LCP}}-A_{\mathrm{RCP}}$$

Chirality is related to the molar ellipticity ([θ]):

2 [θ]=Δε×3298.2

where Δε is the molar CD which is the difference between the molar extinction coefficient of LCP and RCP. Molar ellipticity is generally used to compare chirality values that are measured by different experimental setups. Calculation of molar ellipticity involves the Beer–Lambert law, the details of which are described in Methods.

The CD spectra (in the form of molar ellipticity versus wavelength) of pristine MoS_2_ along with l­(d)-PEN functionalized MoS_2_ are shown in Figure 2a. The CD spectra of l­(d)-PEN only are shown in the inset of Figure 2a. It can be seen that pristine MoS_2_ is chiroptically silent while MoS_2_/l­(d)-PEN samples exhibit molar ellipticity of up to 10^8^ deg·cm^2^/dmol. The ellipticity values of the functionalized 2D MoS_2_ samples are 2–3 orders of magnitude higher than those of 3D chiral materials.^8,10,19,32^ The CD bands of functionalized MoS_2_ are located at ∼300–420 and 420–530 nm. These are substantially different from the CD bands of molecular l­(d)-PEN that are located at 210–270 nm (inset of Figure 2a). In the solid state single-layer MoS_2_ films, we found that the CD bands in the 420–530 nm range show greater amplitude and broadened bandwidth (Figure 2b).

**Figure 2:**
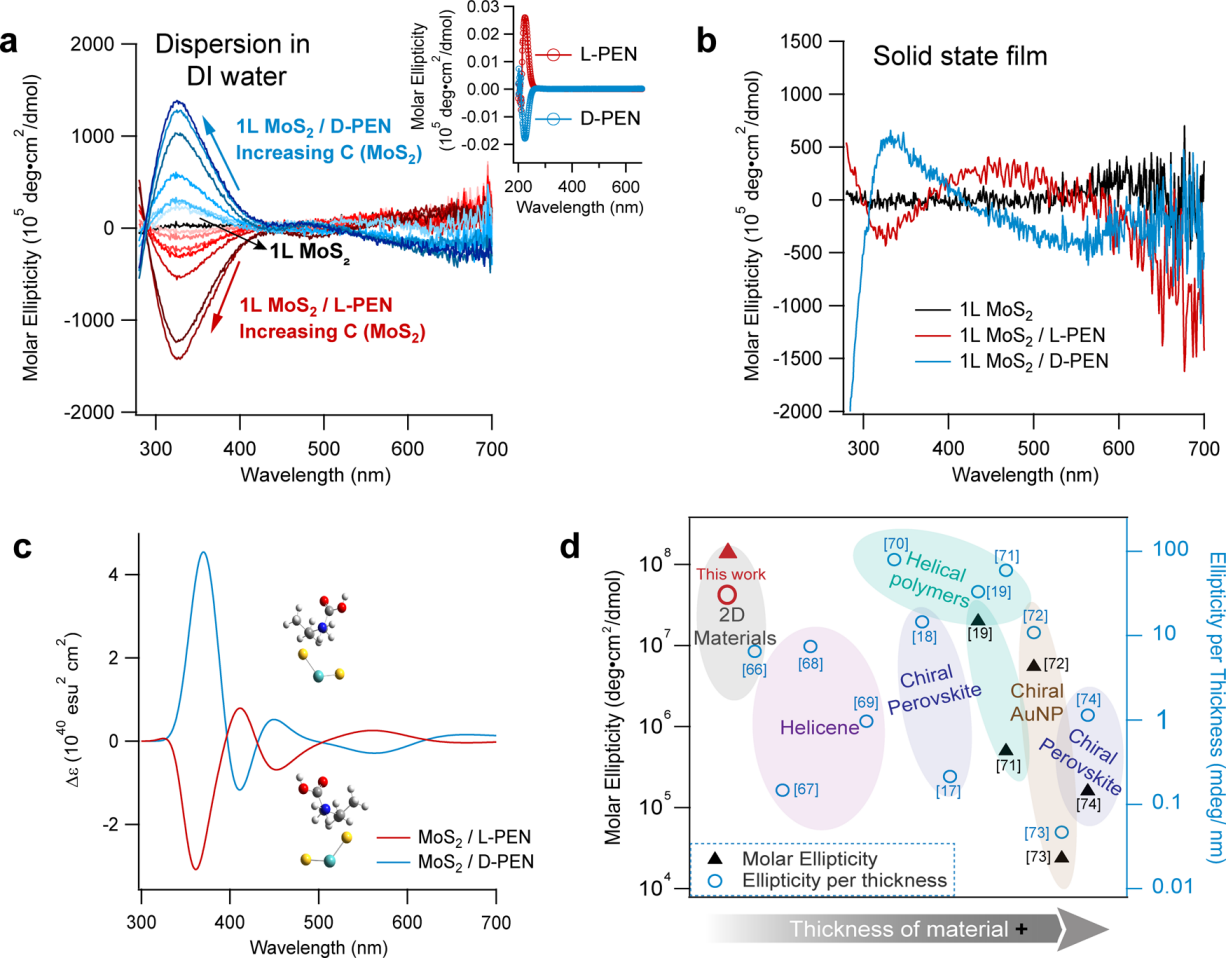
(a) Molar ellipticity of 1L MoS_2_, 1L MoS_2_/l-PEN and 1L MoS_2_/d-PEN as a function of the wavelength. The red and blue arrows indicate chiral-induced CD bands showing higher intensity with the increase of MoS_2_ concentration (denoted as C­(MoS_2_) in panel a). The inset shows the molar ellipticity of molecules only. The broad bands from functionalized MoS_2_ located at ∼300–420 nm and 420–530 nm are absent in pure molecule spectra and pristine MoS_2_. (b) Molar ellipticity of 1L MoS_2_, 1L MoS_2_/l-PEN, and 1L MoS_2_/d-PEN in the solid-state film. The band at ∼300–420 nm is preserved compared to the solution, and the band at ∼420–530 nm is amplified and broadened to ∼400–600 nm. (c) Optimized cluster structures and calculated CD spectra for 1L MoS_2_/l-PEN and 1L MoS_2_/d-PEN. The DFT and TDDFT calculations were performed at the B3LYP/6-31­(d,p) level of theory with solvent effects. (d) Comparison of molar ellipticity and ellipticity per thickness with reported values.^17–19,66–74^

To elucidate the mechanism of chirality in MoS_2_/PEN, we performed density functional theory (DFT) calculations (see Methods). We found that symmetry breaking can be introduced by chemisorption of chiral thiol molecules at S defect sites, giving rise to crystalline distortion (Figures S5 and S6 in the Supporting Information). The calculated CD shown in Figure 2c matches very well with the experimentally measured spectra in Figures 2a,b. In order to cross-check the chemisorption-distortion mechanism, we performed reference experiments with non-thiolated chiral molecules, l/d-valine, to study the role of the thiol group of l­(d)-PEN in imparting chirality to MoS_2_ through chemical interactions. The chemical structure of valine is identical to that of penicillamine with the difference that the headgroup is an H atom instead of −SH. In the absence of the thiol group, we expect l­(d)-valine molecules to be physisorbed on the surface of MoS_2_ and therefore to only weakly perturb the electronic structure or transfer chirality. The CD spectra of MoS_2_ with l­(d)-valine molecules are shown in Figure S7 in the Supporting Information. The CD spectra in Figure S7 do not show additional CD bands from MoS_2_/l­(d)-valine samples even though XPS results clearly show the presence of the molecules on MoS_2_ after functionalization (Figure S8 in the Supporting Information). These results therefore suggest that chemical interactions of sulfur in l­(d)-PEN with MoS_2_ influence adjacent chiral carbon atoms, which leads to structural chirality transfer to MoS_2_. To put our results in context, we compare them with those reported in the literature for solid-state 3D chiral materials in Figure 2d. It can be seen that the absolute molar ellipticity (solid triangles in Figure 2d) of l­(d)-PEN functionalized MoS_2_ is among the highest of any reported material. The molar ellipticity normalized to the thickness is comparable to other materials (hollow circles in Figure 2d), reaching 3.23 mdeg/nm.

The giant molar ellipticity in MoS_2_/PEN suggests strong dissymmetry in light–matter interactions. To further probe how hybridization between PEN and MoS_2_ affects the lattice symmetry and chiroptical properties, we used linearly and circularly polarized Raman spectroscopy.^47^ Since surface functionalization can lead to a change in carrier density that can influence the Raman signal, we first used linearly polarized light to eliminate doping of MoS_2_ as the possible cause of changes in Raman. Monolayer MoS_2_ shows two typical E_2g_^1^ in-plane and A_1g_ out-of-plane Raman vibrational modes [Figure 3a,b] under 532 nm linear laser excitation. After functionalization with l­(d)-PEN, both peaks exhibit broadening and shifts to lower energy, as shown in Figure 3c. We observe a statistically insignificant redshift of the E_2g_^1^ mode of 0.20 ± 0.23 cm^–1^ in MoS_2_/l-PEN and 0.33 ± 0.31 cm^–1^ in MoS_2_/d-PEN. A significant red-shift of 0.87 ± 0.05 cm^–1^ and 1.15 ± 0.22 cm^–1^ is found in the A_1g_ mode of MoS_2_/l-PEN and MoS_2_/d-PEN, respectively. We also compare the full-width half-maximum (fwhm) of the peaks. We observe a negligible change in fwhm of the E_2g_^1^ peak, while the A_1g_ mode fwhm increases from 2.84 ± 0.14 cm^–1^ in the pristine MoS_2_ to 3.36 ± 0.08 cm^–1^ in MoS_2_/l-PEN and 4.43 ± 0.06 cm^–1^ in MoS_2_/d-PEN [Figure 3d]. Since the E_2g_^1^ mode is related to strain due to lattice distortions and A_1g_ mode is affected by electron–phonon coupling caused by doping.^48–50^ The results suggest that functionalized PEN molecules create mostly electronic perturbations in MoS_2_. To evaluate whether these perturbations are chiral, we used circularly polarized excitation in Raman to access symmetry-dependent helicity-resolved phonon scattering. Previous work has reported that pristine 2D TMDs are sensitive to circularly polarized photons, resulting in helicity-resolved Raman modes.^51–53^ The circularly polarized Raman spectra of MoS_2_, MoS_2_/l-PEN, and MoS_2_/d-PEN are plotted in Figure 3e­(i–iii). In both pristine and functionalized MoS_2_, we observe that the peak intensity ratios of E_2g_^1^ and A_1g_ ( $I_{E_{2g}^{1}}/I_{A_{1g}}$ ) are generally higher for excitation by right circularly polarized photons (s+) than for left circularly polarized excitation (s−). The ratio values are listed in Table S1 in the Supporting Information. We observe a significant rise in $I_{E_{2g}^{1}}/I_{A_{1g}}$ in chiral MoS_2_/l-PEN from 0.865 to 0.988 with s+ excitation, while s– excitation remains at a similar value. In contrast, MoS_2_/d-PEN shows higher $I_{E_{2g}^{1}}/I_{A_{1g}}$ with s– excitation, while under s+ excitation, it is unchanged. The Raman optical activity (ROA) is given by

3 $$\mathrm{ROA}=I_{R}-I_{L}$$

where *I*_R_ and *I*_L_ are the intensities of the MoS_2_ Raman peaks when excited by right (s+) and left (s−) circularly polarized light, respectively. The resultant ROA spectra from the three samples plotted in Figure 3f display a negative ROA of −83.4 in E_2g_^1^ and a positive ROA of 140.7 in A_1g_ vibrational modes of pristine MoS_2_. The ROA in MoS_2_/l-PEN shows an enhancement in the E_2g_^1^ to −114.4 and the A_1g_ to 192.2. For MoS_2_/d-PEN, the E_2g_^1^ ROA is nearly tripled to −188.6 whereas an inversion in the sign of A_1g_ ROA of −98.6 is observed. Considering that the measurements are taken on a single flake of monolayer MoS_2_ with a laser excitation spot size of ∼2 mm, the Raman signals are intrinsically weak. However, an ROA intensity of up to 21% of the Raman signal for MoS_2_ indicates high dissymmetry in these phonon modes. The ROA results suggest that functionalization leads to chirality transfer to the monolayer MoS_2_, influencing its vibrational modes.^34^ Together with CD measurements in Figure 2, this demonstrates that the chemical functionalization of 2D MoS_2_ with chiral thiol molecules induces a high order of structural chirality that can interact with circularly polarized light with high efficiency.

**Figure 3:**
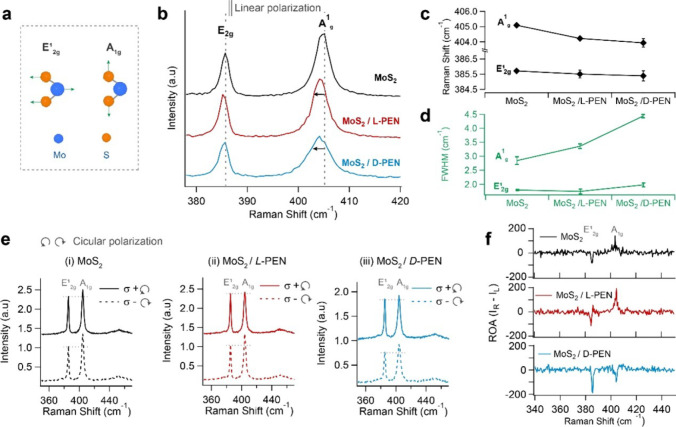
(a) Schematic representation of E_2g_^1^ and A_1g_ Raman vibrational modes of monolayer MoS_2_. (b) Raman spectra of MoS_2_, MoS_2_/l-PEN, and MoS_2_/d-PEN excited with 532 nm linearly polarized light. (c, d) Comparison of (c) peak position and (d) full width at half-maximum (fwhm) of MoS_2_, MoS_2_/l-PEN, and MoS_2_/d-PEN with 532 nm linearly polarized light. (e) Raman spectra of MoS_2_, MoS_2_/l-PEN, and MoS_2_/d-PEN excited with 532 nm left-handed (s+) and right-handed (s−) circularly polarized light. (f) Calculated Raman optical activity (ROA) of MoS_2_, MoS_2_/l-PEN, and MoS_2_/d-PEN.

### Ultrasensitive Detection of Circularly Polarized Photons

The strong chiral light–matter interactions can be used for the photodetection of circularly polarized photons. As 2D MoS_2_ is a direct band gap semiconductor with high incident photon to electron conversion efficiency (IPCE), large asymmetric absorption of left and right circularly polarized (LCP or RCP) photons by chiral 2D MoS_2_/PEN will produce photoelectrons based on the polarization states of incident photons. 2D MoS_2_ has been widely studied as an ultrahigh-gain (>10^3^ A/W) broad-band photodetector (ultraviolet to infrared). However, reports of devices that show sensitivity to circularly polarized light are scarce.^54–57^ We have therefore fabricated phototransistors based on chiral 2D MoS_2_/PEN absorbers as shown schematically in Figure 4a. The gate-dependent photocurrent (*I*_ph_) was monitored by applying a drain–source bias voltage (*V*_ds_) and back-gate voltage (*V*_g_) while illuminating the device with circularly polarized light. The incident photon energy was selected to be 3.06 eV (405 nm), which is close to the peak of the CD band. Pristine MoS_2_ shows strong photodoping as indicated by the increase in current from 10^–12^ A (at *V*_g_ = −30 V) in the dark to 10^–6^ A when illuminated with 178 mW/cm^2^ circularly polarized light. *I*_ph_ values of LCP and RCP overlap because pristine MoS_2_ is achiral [Figure 4b]. For phototransistors with chiral MoS_2_/l-PEN, the dark current remains at 10^–12^ A, but LCP illumination induces a photocurrent of 2.71 × 10^–6^ A, while RCP photons increase it to 1.56 × 10^–9^ A. The giant selection of photon handedness in MoS_2_/l-PEN is consistent with the preferential absorption of LCP over RCP light, meaning that more photons with left circular polarization participate in the excitation process to generate photoelectrons in MoS_2_ as is also evidenced in output curves (*V*_ds_–*I*_ds_) in Figure S9 in the Supporting Information. The asymmetric chiral response is independent of the orientation of electrodes with different crystal edge terminations (Figure S10 in the Supporting Information). We evaluate the efficiency of incident photon to electron conversion by calculating the photoresponsivity (*R*):

4 $$R=\frac{I_{\mathrm{ph}}}{P\times A}$$

where *P* and *A* are the incident light power and the active photodetection area, respectively. Gate-dependent *R* is plotted in Figure S11 in the Supporting Information. *R* of both pristine MoS_2_ and chiral MoS_2_/l-PEN reaches a maximum value of 10^2^ A/W, which is typical for CVD-grown monolayer TMDs.^58–60^ For chiral MoS_2_/l-PEN, *R* of LCP surpasses that of RCP by 2 orders of magnitude, demonstrating large photon to electron conversion anisotropy. The photodetection anisotropy factor (*g*-factor) is defined by

5 $$g=\frac{2\times \left(R_{\mathrm{LCP}}-R_{\mathrm{RCP}}\right)}{R_{\mathrm{LCP}}+R_{\mathrm{RCP}}}$$

where *R*_LCP_ and *R*_RCP_ stands for the *R* of left- and right-circularly polarized light. In the best-performing devices, we measured a *g*-factor of 1.98 at −30 V, which is close to the theoretical upper limit of 2.0 (Figure S12 in the Supporting Information), signifying that the chiral states of photons are fully distinguishable. The average *g*-factor of numerous devices is reported in Figure 4c. Negative *V*_g_ leads to a higher *g*-factor of up to 1.33 ± 0.37 while positive *V*_g_ decreases the *g*-factor to 0.64 ± 0.32. The selective photodetection behavior is also robust, exhibiting reliable cyclability [Figure 4d]. For MoS_2_/d-PEN devices, we observed similar selectivity, albeit with lower photocurrent, as shown in Figure S13 in the Supporting Information. The light power-dependent measurements reveal that the chiral photoresponse is possibly coming from an enhanced/suppressed photogating effect under LCP/RCP illumination, which is analyzed in detail in Figures S14–S16 and the Supporting text in the Supporting Information. At 642 nm, where the CD band of MoS_2_/l-PEN is negative, the photocurrent of RCP surpasses LCP, which is demonstrated in Figure S17 in the Supporting Information. We therefore conclude that chiral MoS_2_/l-PEN devices are highly selective, sensitive, and responsive photodetectors for circularly polarized light compared to devices from 3D materials despite MoS_2_/l-PEN being only 1 nm thick [Figure 4e].

**Figure 4:**
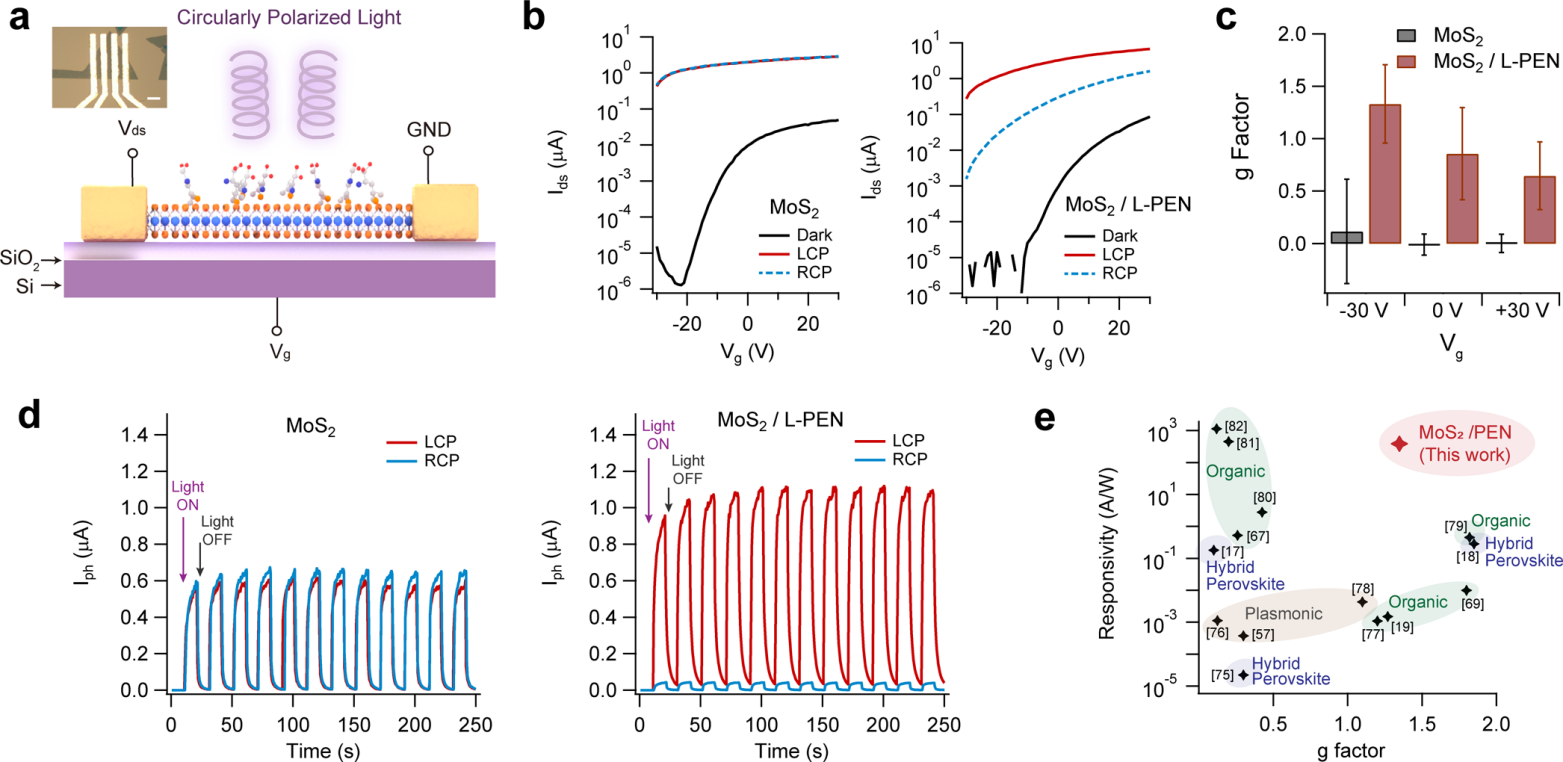
(a) Device structure of phototransistor for circularly polarized light detection. The inset shows an optical microscope photo of a typical measured device. The scale bar is 10 mm. (b) Gate-dependent photoresponse MoS_2_ and MoS_2_/l-PEN illuminated by left and right circularly polarized light (LCP and RCP). (c) Calculated *g*-factor of photoresponsivity of MoS_2_ and MoS_2_/l-PEN phototransistor under different gate bias. (d) Time-dependent photoresponse of MoS_2_ and MoS_2_/l-PEN under *V*_g_ = 0 V and *V*_ds_ = 1 V bias. (e) Comparison of photoresponsivity as a function of *g*-factor of MoS_2_/PEN with reported values.^17–19,57,67,69,75–82^

## Conclusions

In summary, we demonstrate chirality transfer in 2D MoS_2_ by chemical functionalization with chiral thiol-based molecules. The sub-nanometer thick functionalized monolayer exhibits ultrahigh molar ellipticity mediated by the chemisorbed chiral thiol. The chiral molecule imparts a local atomic chiral field that influences the interactions between the 2D TMD and incident photons. The strong chirality-selective light–matter interactions give rise to regulation of the photoinduced electrons in the semiconducting MoS_2_. The high molar ellipticity in chemically activated chiral 2D MoS_2_ can be used for highly efficient detection of circularly polarized photons.

## Methods

### Preparation of Single Layer MoS_2_

To obtain symmetric CD spectra, we used MoS_2_ with a high amount of sulfur vacancies to allow ample functionalization by both l-PEN and d-PEN molecules, without sacrificing the optical and electronic properties. We did this by obtaining uniform films of monolayer MoS_2_ by chemical vapor and exfoliation methods.

### CVD Growth of Monolayer MoS_2_

A single-zone tube furnace was used for the growth. 2.5 mg of MoO_3_ powder was evenly distributed in an alumina boat and located at the center of the furnace. A layer of molecular sieves (Alfa Aesar 5A 1–2 mm diameter pellets) covered the MoO_3_ boat to control the growth rate. SiO_2_/Si substrates, spin-coated with 0.5 mg/mL NaOH promoter, were placed on the MoO_3_ boat. 60 mg of sulfur powder in another alumina boat was located 17 cm upstream at the edge of the furnace. Before starting growth, the tube was purged with 460 sccm N_2_ for 20 min. Then the N_2_ flow rate was decreased to 60 sccm. The temperature of MoO_3_ was set and kept at 720 °C for 10 min, while the temperature of sulfur was allowed to reach 230 °C. After growth, the furnace was switched off, and the furnace was cooled down in 460 sccm N_2_ gas.

### Chemical Functionalization of MoS_2_

Commercially available l-PEN, l-valine, and d-valine were purchased from Merck. d-PEN was purchased from Cambridge Biotech. 7.6 mmol of the chiral molecule and 1L MoS_2_ were either dispersed in DI water or transferred onto a substrate and placed in a sealed container. For functionalization in an aqueous environment, the molecular concentration was fixed at 2 g/L. A mother solution of 1L MoS_2_ was diluted in a series of concentrations, ranging from 8 mg/L to 200 mg/L. The mixture was stirred at room temperature for 24 h followed by discarding the unreacted precipitate. For functionalization in a vapor environment, to create a thiol vapor environment without decomposing the molecule, the beaker was first heated to 450 K, kept at this temperature for 20 min, then heated to 480 K and kept there for 20 min, then finally heated to 500 K and held for 4 h before slowly cooling down to room temperature. The samples were taken out from the sealed container and placed on the hot plate at 500 K in a N_2_-filled glovebox for at least 20 min to maximally desorb physisorbed molecules. This was all carried out in a N_2_ filled glovebox with H_2_O < 1 ppm and O_2_ < 5 ppm.

### AFM Measurements

AFM imaging was performed by means of a Bruker Dimension Icon operating in airPeakForce Tapping (with ScanAsyst), by using tip model SCANASYST-AIR (tip stiffness: *k* = 0.4 N/m).

### X-ray Absorption Spectroscopy

The X-ray absorption spectroscopy (XAS) was measured at Beamline I09 at the Diamond Light Source (Oxford, UK). For XAS measurements, CVD-grown MoS_2_ films were transferred to Pt-coated Si/SiO_2_ substrates and were loaded in a vacuum chamber at 10^–10^ mbar. The incident X-ray beam was 70° with respect to the normal axis to the basal plane. The beam spot size was approximately 300 mm.

### X-ray Photoelectron Spectroscopy

X-ray Photoelectron Spectroscopy (XPS) measurements were carried out with a Thermo Fisher Nexsa-G2 spectrometer with Cu Kα source on CVD-grown monolayer materials on Si/SiO_2_ (oxide thickness = 90 nm). The spot size was 50–100 mm, which can cover the region of a single flake of CVD-grown MoS_2_. A modified flood gun compensation mode was employed to avoid damage to the organic molecule and charging on the nonconductive sample to accurately analyze the chemical functionalization.

### Circular Dichroism (CD)

To measure the CD of a single-layer MoS_2_ film, we dry-transferred the as-grown film assisted by UV–ozone-treated PDMS onto the inner wall of a strain-free quartz cuvette to maximally eliminate the linear dichroism effect. CD spectra were recorded with the Aviv 410 CD and Chirascan V100 spectrometer. The obtained CD spectra from the spectrometer were originally in ellipticity (θ in mdeg) units, which is related to the electrical field vector of LCP (*E*_L_) and RCP (*E*_R_), where

$$\tan\theta =\frac{\left(E_{R}-E_{L}\right)}{\left(E_{R}+E_{L}\right)}\approx \theta$$

Considering that the intensity of light is proportional to the square of the electrical field vector, converting the radian value to degrees, we obtain

$$\theta =\Delta A\left(\frac{\ln10}{4}\right)\left(\frac{180}{\pi }\right)\approx \Delta A\times 32.982$$

According to the Beer–Lambert law, Δ*A* = Δε*lc*, by normalizing the ellipticity with light path length *l* and molar concentration *c* and the average molecular weight *M*, we obtain molar ellipticity expressed as

$$\left[\theta \right]=\frac{\theta M}{10lc}$$

The units of [θ] are deg·cm^2^·dmol^–1^. For monolayer MoS_2_, we define

$${\left[\theta \right]}_{\mathrm{Mo}S_{2}}=\frac{\theta_{\mathrm{Mo}S_{2}}M_{\mathrm{Mo}S_{2}}}{10lc}=\frac{\theta_{\mathrm{Mo}S_{2}}M_{\mathrm{Mo}S_{2}}}{10{m_{\mathrm{Mo}S_{2}}}^{\times }A_{\mathrm{Mo}S_{2}}}$$

The mass (*m*_MoS_2__) of MoS_2_ in a typical area (*A*_MoS_2__) of 10 mm × 8 mm film for CD measurement can be estimated by considering a unit cell for monolayer MoS_2_ being 3.17 Å × 5.5 Å, consisting of 2 Mo and 4 S atoms. Assuming 100% material coverage, the obtained *m*_MoS_2__ value is 0.24396 mg. For MoS_2_/PEN, we take approximately 60% degree of functionalization for both physisorbed and chemisorbed molecules; the obtained *m*_MoS_2_/PEN_ is 0.24396 + 0.002473 mg ≈ *m*_MoS_2__.

### UV–Vis Absorption, Reflectance, and Transmission

UV–vis spectra were measured with an Agilent Cary 7000 spectrometer. CVD samples were transferred onto a quartz substrate (Agar Scientific) and stabilized on a sample holder with an 8 × 8 mm^2^ aperture size.

### Device Fabrication

For device fabrication, CVD MoS_2_ was first transferred onto thermally oxidized heavily n-doped silicon substrates (*t*_ox_ = 90 nm). Top electrodes were patterned with photolithography (laser writer LW405B from Microtech, AZ5214E photoresist) followed by metal deposition (10 nm In/80 nm Au) and the lift-off process. The device was annealed under vacuum (10^–6^ Torr) for 10 h before measurement. The chemical functionalization (see Chemical functionalization of MoS_2_ in Methods) was carried out after devices were fabricated and tested to ensure they were operational (noted as “MoS_2_” in Figure 4).

### Electrical Characterization

The characterization of device performance was realized by a customized probe station equipped with a Keithley 2612 SMU in ambient atmosphere. The monochromic laser was produced by a fiber-coupled Laser Source (Thorlabs MCLS1). Polarization of light was done with a linear polarizer (WP25M-VIS) and a quarter-waveplate (WPMQ05M-405) before reaching the sample through the optical lens. The output power was calibrated by a PM400 Power Meter (Thorlabs). The spot size was around 50 mm^2^.

### Raman Spectroscopy

Raman and photoluminescence spectra were measured using a Horiba Evolution spectrometer equipped with a 532 nm laser. The excitation power was kept below 1 mW to avoid local heat damage effects. The wavenumber (energy) resolution was ∼1 meV. For circularly polarized Raman, a customized optical setup was utilized. The detailed optical setup can be found in Figure S18 in the Supporting Information.

### Density Functional Theory (DFT) Calculations

For the periodic crystal structures of MoS_2_/l-PEN and MoS_2_/d-PEN, DFT calculations were performed using the projector-augmented wave (PAW) method,^61^ as implemented in the Vienna Ab Initio Simulation Package (VASP).^62,63^ The generalized gradient approximation (GGA) with the Perdew–Burke–Ernzerhof (PBE) exchange correlation functional was employed.^64^ The van der Waals (vdW) interactions were included during structural optimization through the zero-damping method of Grimme (DFT-D3).^65^ A 5 × 5 1T-MoS_2_ supercell was used to study the molecular adsorption of PEN molecules. The energy cutoffs of wave functions were set to 520 eV, and a 2 × 2 × 1 *k*-point mesh with Gaussian smearing of 0.05 eV was used. All crystal structures were fully relaxed until the Hellmann–Feynman forces on each atom were less than 0.01 eV/Å.

For the cluster structures of 1L MoS_2_/l-PEN and 1L MoS_2_/d-PEN, ground-state geometries were optimized using the B3LYP functional with the 6-31G­(d,p) basis set. Time-dependent DFT (TDDFT) calculations were performed to obtain the circular dichroism (CD) spectra, including excitation energies and corresponding oscillator strengths. The solvent effect of water (ε = 78.36) was taken into consideration in structural optimizations and optical calculations using the conductor-like polarizable continuum model (CPCM). All the cluster calculations were carried out using the Gaussian16 program.

## Supplementary Material



## Notes

Figure S1. XPS spectra of MoS_2_ functionalized with l/d-PEN; Figure S2. AFM morphologies of MoS_2_, MoS_2_/l-PEN and MoS_2_/d-PEN; Figure S3. FTIR spectrum of MoS_2_, PEN and MoS_2_/PEN; Figure S4. S K-edge XANES spectra of MoS_2_, MoS_2_/l-PEN and MoS_2_/d-PEN; Figure S5. Collective distortion by DFT calculation; Figure S6. Optimized crystal structures and electronic bands structures; Figure S7. Molar ellipticity of CVD grown monolayer MoS_2_, MoS_2_/l-valine and MoS_2_/d-valine; Figure S8. Core-level Mo 3d, S 2s, C 1s, and N 1s XPS spectra of MoS_2_, MoS_2_/l-valine and MoS_2_/d-valine; Table S1. E_2g_^1^ and A_1g_ Raman peak intensity ratio; Figure S9. Output characteristics of MoS_2_ and MoS_2_/l-PEN phototransistors; Figure S10. Effect of orientation of electrodes; Figure S11. Gate-dependent photoresponsivity; Figure S12. Gate-dependent g factor; Figure S13. Gate-dependent photoresponse; Supporting Text: Power-dependent photoresponse of MoS_2_ and MoS_2_/l-PEN phototransistors; Figure S14. Power-dependent photoresponse; Figure S15. Calculation and analysis of power-dependent chiral responsivity; Figure S16. Power-dependent specific detectivity; Figure S17. Photodetection of circularly polarized light at 642 nm. Figure S18. Optical setup of Raman optical activity measurement (PDF)

## References

[1] Gal J. (2017). Pasteur and
the art of chirality. Nature Chem..

[2] Wan L., Shi X. Y., Wade J., Campbell A. J., Fuchter M. J. (2021). Strongly
Circularly Polarized Crystalline and β-Phase Emission from Poly­(9,9-dioctylfluorene)-Based
Deep-Blue Light-Emitting Diodes. Adv. Opt Mater..

[3] Ma B. J., Bianco A. (2023). Regulation of biological processes by intrinsically
chiral engineered materials. Nat. Rev. Mater..

[4] Yoon T. P., Jacobsen E. N. (2003). Privileged
chiral catalysts. Science.

[5] Lodahl P., Mahmoodian S., Stobbe S., Rauschenbeutel A., Schneeweiss P., Volz J., Pichler H., Zoller P. (2017). Chiral quantum
optics. Nature.

[6] Söllner I., Mahmoodian S., Hansen S. L., Midolo L., Javadi A., Kiršanskė G., Pregnolato T., El-Ella H., Lee E. H., Song J. D. (2015). Deterministic
photon–emitter coupling in chiral photonic circuits. Nature Nanotechnol..

[7] Cheong S.-W., Xu X. (2022). Magnetic chirality. npj Quantum Materials.

[8] Long G. K., Sabatini R., Saidaminov M. I., Lakhwani G., Rasmita A., Liu X. G., Sargent E. H., Gao W. B. (2020). Chiral-perovskite
optoelectronics. Nat. Rev. Mater..

[9] Naaman R., Paltiel Y., Waldeck D. H. (2019). Chiral
molecules and the electron
spin. Nat. Rev. Chem..

[10] Yeom J., Santos U. S., Chekini M., Cha M., de Moura A. F., Kotov N. A. (2018). Chiromagnetic nanoparticles and gels. Science.

[11] Crassous J., Fuchter M. J., Freedman D. E., Kotov N. A., Moon J., Beard M. C., Feldmann S. (2023). Materials for chiral light control. Nat. Rev. Mater..

[12] Jiang S., Kotov N. A. (2023). Circular polarized
light emission in chiral inorganic
nanomaterials. Adv. Mater..

[13] Chen S., Katsis D., Schmid A., Mastrangelo J., Tsutsui T., Blanton T. (1999). Circularly polarized
light generated
by photoexcitation of luminophores in glassy liquid-crystal films. Nature.

[14] Kumar J., Nakashima T., Kawai T. (2015). Circularly polarized luminescence
in chiral molecules and supramolecular assemblies. journal of physical chemistry letters.

[15] Kim Y.-H., Zhai Y., Lu H., Pan X., Xiao C., Gaulding E. A., Harvey S. P., Berry J. J., Vardeny Z. V., Luther J. M., Beard M. C. (2021). Chiral-induced spin
selectivity enables
a room-temperature spin light-emitting diode. Science.

[16] Ji Z., Liu W., Krylyuk S., Fan X., Zhang Z., Pan A., Feng L., Davydov A., Agarwal R. (2020). Photocurrent detection
of the orbital angular momentum of light. Science.

[17] Chen C., Gao L., Gao W., Ge C., Du X., Li Z., Yang Y., Niu G., Tang J. (2019). Circularly polarized
light detection using chiral hybrid perovskite. Nat. Commun..

[18] Ishii A., Miyasaka T. (2020). Direct detection of circular polarized light in helical
1D perovskite-based photodiode. Science advances.

[19] Song I. H., Ahn J., Ahn H., Lee S. H., Mei J. G., Kotov N. A., Oh J. H. (2023). Helical polymers for dissymmetric circularly polarized light imaging. Nature.

[20] Wang Q. H., Kalantar-Zadeh K., Kis A., Coleman J. N., Strano M. S. (2012). Electronics
and optoelectronics of two-dimensional transition metal dichalcogenides. Nature Nanotechnol..

[21] Manzeli S., Ovchinnikov D., Pasquier D., Yazyev O. V., Kis A. (2017). 2D transition
metal dichalcogenides. Nat. Rev. Mater..

[22] Splendiani A., Sun L., Zhang Y., Li T., Kim J., Chim C.-Y., Galli G., Wang F. (2010). Emerging photoluminescence
in monolayer
MoS2. Nano Lett..

[23] Eda G., Yamaguchi H., Voiry D., Fujita T., Chen M., Chhowalla M. (2011). Photoluminescence
from chemically exfoliated MoS2. Nano Lett..

[24] Koperski M., Nogajewski K., Arora A., Cherkez V., Mallet P., Veuillen J.-Y., Marcus J., Kossacki P., Potemski M. (2015). Single photon
emitters in exfoliated WSe2 structures. Nature
Nanotechnol..

[25] Srivastava A., Sidler M., Allain A. V., Lembke D. S., Kis A., Imamoğlu A. (2015). Optically active quantum dots in monolayer WSe2. Nature Nanotechnol..

[26] Radisavljevic B., Radenovic A., Brivio J., Giacometti V., Kis A. (2011). Single-layer MoS2 transistors. Nature Nanotechnol..

[27] Lembke D., Kis A. (2012). Breakdown of high-performance
monolayer MoS2 transistors. ACS Nano.

[28] Chang H.-Y., Yang S., Lee J., Tao L., Hwang W.-S., Jena D., Lu N., Akinwande D. (2013). High-performance,
highly bendable MoS2 transistors with high-k dielectrics for flexible
low-power systems. ACS Nano.

[29] Liu T., Liu S., Tu K.-H., Schmidt H., Chu L., Xiang D., Martin J., Eda G., Ross C. A., Garaj S. (2019). Crested two-dimensional
transistors. Nature Nanotechnol..

[30] Giaconi N., Poggini L., Lupi M., Briganti M., Kumar A., Das T. K., Sorrentino A. L., Viglianisi C., Menichetti S., Naaman R. (2023). Efficient
Spin-Selective
Electron Transport at Low Voltages of Thia-Bridged Triarylamine Hetero[4]­helicenes
Chemisorbed Monolayer. ACS Nano.

[31] Chernikov, A. ; Berkelbach, T. C. ; Hill, H. M. ; Rigosi, A. ; Li, Y. ; Aslan, Ö. B. ; Reichman, D. R. ; Hybertsen, M. S. ; Heinz, T. F. Excitons in atomically thin transition-metal dichalcogenides. In 2014 Conference on Lasers and Electro-Optics (CLEO)-Laser Science to Photonic Applications; IEEE: 2014; pp 1–2.

[32] Kim R. M., Huh J. H., Yoo S., Kim T. G., Kim C., Kim H., Han J. H., Cho N. H., Lim Y. C., Im S. W. (2022). Enantioselective sensing by collective circular dichroism. Nature.

[33] Kumar P., Vo T., Cha M. J., Visheratina A., Kim J. Y., Xu W. Q., Schwartz J., Simon A., Katz D., Nicu V. P. (2023). Photonically active
bowtie nanoassemblies with chirality continuum. Nature.

[34] Qian Q., Ren H., Zhou J., Wan Z., Zhou J., Yan X., Cai J., Wang P., Li B., Sofer Z. (2022). Chiral
molecular intercalation superlattices. Nature.

[35] Di
Nuzzo D., Cui L., Greenfield J. L., Zhao B., Friend R. H., Meskers S. C. (2020). Circularly polarized
photoluminescence from chiral perovskite thin films at room temperature. ACS Nano.

[36] Purcell-Milton F., McKenna R., Brennan L. J., Cullen C. P., Guillemeney L., Tepliakov N. V., Baimuratov A. S., Rukhlenko I. D., Perova T. S., Duesberg G. S. (2018). Induction of Chirality
in Two-Dimensional Nanomaterials: Chiral 2D MoS(2) Nanostructures. ACS Nano.

[37] Castner D. G., Hinds K., Grainger D. W. (1996). X-ray photoelectron spectroscopy
sulfur 2p study of organic thiol and disulfide binding interactions
with gold surfaces. Langmuir.

[38] Nguyen E. P., Carey B. J., Ou J. Z., van Embden J., Della Gaspera E., Chrimes A. F., Spencer M. J. S., Zhuiykov S., Kalantar-zadeh K., Daeneke T. (2015). Electronic Tuning of
2D MoS2 through
Surface Functionalization. Adv. Mater..

[39] Ding Q., Czech K. J., Zhao Y. Z., Zhai J. Y., Hamers R. J., Wright J. C., Jin S. (2017). Basal-Plane Ligand Functionalization
on Semiconducting 2H-MoS2Monolayers. Acs Appl.
Mater. Inter.

[40] Li D., Bancroft G. M., Kasrai M., Fleet M. E., Feng X. H., Tan K. H. (1995). Polarized X-Ray-Absorption Spectra and Electronic-Structure
of Molybdenite (2h-Mos2). Phys. Chem. Miner.

[41] Bertolazzi S., Bonacchi S., Nan G. J., Pershin A., Beljonne D., Samori P. (2017). Engineering
Chemically Active Defects in Monolayer
MoS2 Transistors via Ion-Beam Irradiation and Their Healing via Vapor
Deposition of Alkanethiols. Adv. Mater..

[42] Park S., Garcia-Esparza A. T., Abroshan H., Abraham B., Vinson J., Gallo A., Nordlund D., Park J., Kim T. R., Vallez L. (2021). Operando Study of Thermal Oxidation of Monolayer MoS. Adv. Sci..

[43] Yang R., Morris D. J., Higaki T., Ward M. J., Jin R. C., Zhang P. (2018). Sensitive X-ray Absorption Near Edge Structure Analysis on the Bonding
Properties of Au (SR) Nanoclusters. Acs Omega.

[44] Guay D., Divigalpitiya W. M. R., Belanger D., Feng X. H. (1994). Chemical Bonding
in Restacked Single-Layer Mos(2) by X-Ray-Absorption Spectroscopy. Chem. Mater..

[45] Lee M., Kim Y., Mohamed A. Y., Lee H. K., Ihm K., Kim D. H., Park T. J., Cho D. Y. (2020). Direct Evidence of Electronic Interaction
at the Atomic-Layer-Deposited MoS Monolayer/SiO Interface. Acs Appl. Mater. Inter.

[46] Rompel A., Cinco R. M., Latimer M. J., McDermott A. E., Guiles R. D., Quintanilha A., Krauss R. M., Sauer K., Yachandra V. K., Klein M. P. (1998). Sulfur K-edge x-ray absorption spectroscopy::
A spectroscopic tool to examine the redox state of S-containing metabolites. P Natl. Acad. Sci. USA.

[47] Zhou K. G., Withers F., Cao Y., Hu S., Yu G. L., Casiraghi C. (2014). Raman Modes of MoS Used as Fingerprint
of van der Waals
Interactions in 2-D Crystal-Based Heterostructures. ACS Nano.

[48] Chakraborty B., Bera A., Muthu D. V. S., Bhowmick S., Waghmare U. V., Sood A. K. (2012). Symmetry-dependent
phonon renormalization in monolayer
MoS transistor. Phys. Rev. B.

[49] Li Y., Xu C. Y., Hu P. A., Zhen L. (2013). Carrier Control of
MoS Nanoflakes by Functional Self-Assembled Monolayers. ACS Nano.

[50] Velicky M., Rodriguez A., Bousa M., Krayev A. V., Vondrácek M., Honolka J., Ahmadi M., Donnelly G. E., Huang F. M., Abruña H. D. (2020). Strain and Charge Doping Fingerprints
of the Strong Interaction between Monolayer MoS and Gold. J. Phys. Chem. Lett..

[51] Chen S. Y., Zheng C. X., Fuhrer M. S., Yan J. (2015). Helicity-Resolved Raman
Scattering of MoS2, MoSe2, WS2, and WSe2 Atomic Layers. Nano Lett..

[52] Drapcho S. G., Kim J., Hong X. P., Jin C. H., Shi S. F., Tongay S., Wu J. Q., Wang F. (2017). Apparent breakdown of Raman selection
rule at valley exciton resonances in monolayer MoS. Phys. Rev. B.

[53] Zhao Y., Zhang S. S., Shi Y. P., Zhang Y. F., Saito R., Zhang J., Tong L. M. (2020). Characterization
of Excitonic Nature
in Raman Spectra Using Circularly Polarized Light. ACS Nano.

[54] Lopez-Sanchez O., Lembke D., Kayci M., Radenovic A., Kis A. (2013). Ultrasensitive photodetectors based
on monolayer MoS2. Nat. Nanotechnol..

[55] Kufer D., Konstantatos G. (2015). Highly Sensitive, Encapsulated MoS2 Photodetector with
Gate Controllable Gain and Speed. Nano Lett..

[56] Xin W., Zhong W., Shi Y., Shi Y., Jing J., Xu T., Guo J., Liu W., Li Y., Liang Z. (2024). Low-dimensional Materials-Based
Photodetectors for Next-Generation
Polarized Detection And Imaging. Adv. Mater..

[57] Bu Y. H., Ren X. S., Zhou J., Zhang Z. H., Deng J., Xu H. Y., Xie R. Z., Li T. X., Hu W. D., Guo X. (2023). Configurable
circular-polarization-dependent optoelectronic
silent state for ultrahigh light ellipticity discrimination. Light-Sci. Appl..

[58] George A., Fistul M. V., Gruenewald M., Kaiser D., Lehnert T., Mupparapu R., Neumann C., Hubner U., Schaal M., Masurkar N. (2021). Giant persistent photoconductivity in monolayer
MoS2 field-effect transistors. Npj 2d Mater.
Appl..

[59] Li S. Y., Chen X. Q., Liu F. M., Chen Y. F., Liu B. Y., Deng W. J., An B. X., Chu F. H., Zhang G. Q., Li S. L. (2019). Enhanced Performance
of a CVD MoS2 Photodetector by
Chemical in Situ n-Type Doping. Acs Appl. Mater.
Inter.

[60] Hoang A. T., Hu L. H., Kim B. J., Van T. T. N., Park K. D., Jeong Y., Lee K., Ji S., Hong J., Katiyar A. K. (2023). Low-temperature growth of MoS2 on polymer and
thin glass substrates for flexible electronics. Nat. Nanotechnol..

[61] Blochl P. E. (1994). Projector
augmented-wave method. Phys. Rev. B Condens
Matter.

[62] Kresse G., Furthmüller J. (1996). Efficiency of ab-initio total energy
calculations for
metals and semiconductors using a plane-wave basis set. Computational materials science.

[63] Kresse G., Hafner J. (1993). Ab initio molecular
dynamics for liquid metals. Phys. Rev. B.

[64] Perdew J. P., Burke K., Ernzerhof M. (1996). Generalized
gradient approximation
made simple. Physical review letters.

[65] Grimme S., Ehrlich S., Goerigk L. (2011). Effect of
the damping function in
dispersion corrected density functional theory. Journal of computational chemistry.

[66] Kim C. J., Sánchez-Castillo A., Ziegler Z., Ogawa Y., Noguez C., Park J. (2016). Chiral atomically
thin films. Nat. Nanotechnol..

[67] Zhu D. L., Jiang W., Ma Z. T., Feng J. J., Zhan X. Q., Lu C., Liu J., Hu Y. Y., Wang D., Zhao Y. S. (2022). Organic
donor-acceptor heterojunctions for high performance circularly
polarized light detection. Nat. Commun..

[68] Wade J., Salerno F., Kilbride R. C., Kim D. K., Schmidt J. A., Smith J. A., LeBlanc L. M., Wolpert E. H., Adeleke A. A., Johnson E. R. (2022). Controlling anisotropic properties by manipulating
the orientation of chiral small molecules. Nat.
Chem..

[69] Yang Y., da Costa R. C., Fuchter M. J., Campbell A. J. (2013). Circularly polarized
light detection by a chiral organic semiconductor transistor. Nat. Photonics.

[70] Wade J., Hilfiker J. N., Brandt J. R., Liirò-Peluso L., Wan L., Shi X. Y., Salerno F., Ryan S. T. J., Schöche S., Arteaga O. (2020). Natural
optical activity as the origin of the large
chiroptical properties in π-conjugated polymer thin films. Nat. Commun..

[71] Zhang H. K., Zheng X. Y., Kwok R. T. K., Wang J., Leung N. L. C., Shi L., Sun J. Z., Tang Z. Y., Lam J. W. Y., Qin A. J., Tang B. Z. (2018). In situ monitoring
of molecular aggregation
using circular dichroism. Nat. Commun..

[72] Xu L. G., Wang X. X., Wang W. W., Sun M. Z., Choi W. J., Kim J. Y., Hao C. L., Li S., Qu A. H., Lu M. R. (2022). Enantiomer-dependent
immunological response to chiral
nanoparticles. Nature.

[73] Zheng J. P., Boukouvala C., Lewis G. R., Ma Y. C., Chen Y., Ringe E., Shao L., Huang Z. F., Wang J. F. (2023). Halide-assisted
differential growth of chiral nanoparticles with threefold rotational
symmetry. Nat. Commun..

[74] Hu Y., Florio F., Chen Z. Z., Phelan W. A., Siegler M. A., Zhou Z., Guo Y. W., Hawks R., Jiang J., Feng J. (2020). A chiral
switchable photovoltaic ferroelectric 1D perovskite. Science Advances.

[75] Li D., Liu X. T., Wu W. T., Peng Y., Zhao S. G., Li L. N., Hong M. C., Luo J. H. (2021). Chiral Lead-Free
Hybrid Perovskites for Self-Powered Circularly Polarized Light Detection. Angew. Chem. Int. Edit.

[76] Cai J. R., Zhang W., Xu L. G., Hao C. L., Ma W., Sun M. Z., Wu X. L., Qin X., Colombari F. M., de Moura A. F. (2022). Polarization-sensitive
optoionic membranes
from chiral plasmonic nanoparticles. Nat. Nanotechnol..

[77] Shi W. D., Salerno F., Ward M. D., Santana-Bonilla A., Wade J., Hou X. Y., Liu T., Dennis T. J. S., Campbell A. J., Jelfs K. E., Fuchter M. J. (2021). Fullerene
Desymmetrization
as a Means to Achieve Single-Enantiomer Electron Acceptors with Maximized
Chiroptical Responsiveness. Adv. Mater..

[78] Li W., Coppens Z. J., Besteiro L. V., Wang W. Y., Govorov A. O., Valentine J. (2015). Circularly polarized light detection with hot electrons
in chiral plasmonic metamaterials. Nat. Commun..

[79] Gao Y. R., Liao J. W., Chen H. Y., Ning H. J., Wu Q. H., Li Z. L., Wang Z. Y., Zhang X. L., Shao M., Yu Y. (2023). High Performance Polarization-Resolved
Photodetectors Based on Intrinsically
Stretchable Organic Semiconductors. Adv. Sci..

[80] Zhang C., Xu C. Y., Chen C. F., Cheng J. J., Zhang H. L., Ni F., Wang X. H., Zou G., Qiu L. Z. (2022). Optically Programmable
Circularly Polarized Photodetector. ACS Nano.

[81] Hao J., Lu H. P., Mao L. L., Chen X. H., Beard M. C., Blackburn J. L. (2021). Direct
Detection of Circularly Polarized Light Using
Chiral Copper Chloride-Carbon Nanotube Heterostructures. ACS Nano.

[82] Shang X., Song I., Lee J. H., Choi W., Ahn J., Ohtsu H., Kim J. C., Koo J. Y., Kwak S. K., Oh J. H. (2020). Surface-Doped Quasi-2D
Chiral Organic Single Crystals for Chiroptical
Sensing. ACS Nano.

