# Supplementary material for: Ultrasensitive Circularly Polarized Photon Detectors Based on Chiral Two-Dimensional MoS2
Source: ACS Nano. 2025 Oct 23;19(43):37919–28. doi: 10.1021/acsnano.5c12182 (PMC12593354; doi:10.1021/acsnano.5c12182)
Supplement: Supplementary file 1 [file nn5c12182_si_001.pdf]

Supporting Information for

Ultra-sensitive circularly polarized photon detectors based on chiral two-dimensional MoS_2

Ye Wang[1]*, Yiru Zhu[1], Tieyuan Bian[2], Ziwei Jeffery Yang[1], Yuanyuan Zhao[3], Han Yan[1], Yang Li[1], Yan Wang[1], Feng Ding[3,4], Jun Yin[2], Manish Chhowalla[1]*

[1]Department of Materials Science & Metallurgy, University of Cambridge; 27 Charles Babbage Road, Cambridge CB3 0FS, United Kingdom.
[2]Department of Applied Physics, The Hong Kong Polytechnic University; Kowloon, Hong Kong.
[3]Institute of Technology for Carbon Neutrality, Shenzhen Institute of Advanced Technology, Chinese Academy of Sciences, Shenzhen, China.
[4]Suzhou Laboratory; Suzhou, 215123 China.

*Corresponding author. Manish Chhowalla mc209@cam.ac.uk ; Ye Wang y.wang19@tue.nl

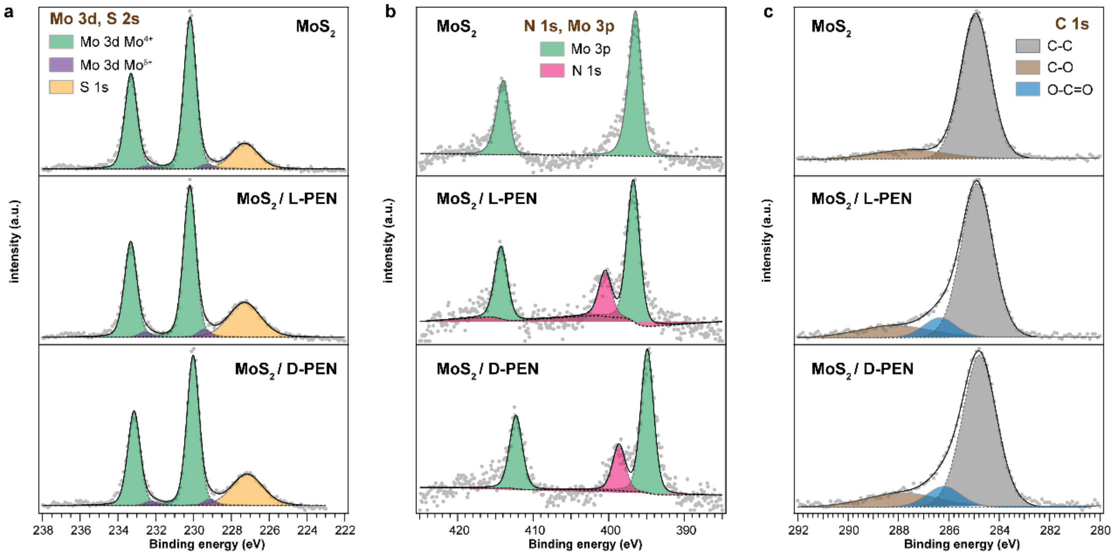

Fig. S1. XPS spectra of MoS_2 functionalized with L/D-PEN. (a) Core-level Mo 3d, S 1s XPS spectra of MoS_2, MoS_2/L-PEN and MoS_2/D-PEN. (b) Core-level N 1s, Mo 3p XPS spectra of MoS_2, MoS_2/L-PEN and MoS_2/D-PEN indicating the presence of amine group ($-NH_2$) after molecular functionalization. (c) Core-level C 1s and S 2p XPS spectra of MoS_2, MoS_2/L-PEN and MoS_2/D-PEN showing appearance of C-O components. These evidences that signatures groups of the molecules are present on are functionalized on the surfaces of MoS_2.

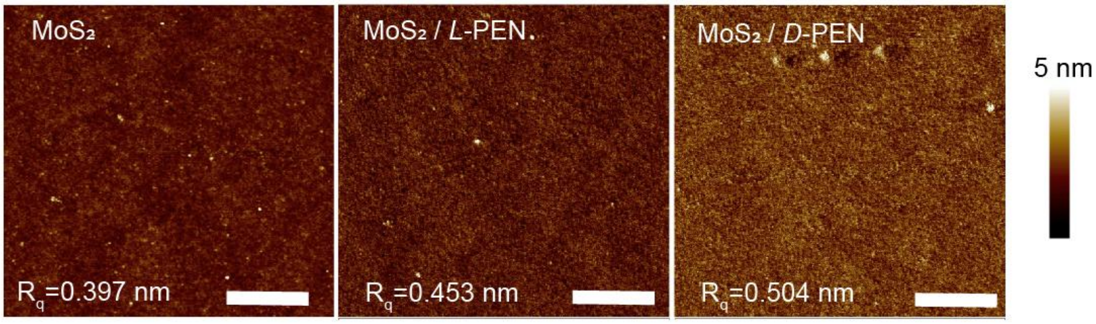

Fig. S2. AFM morphologies of MoS_2, MoS_2/L-PEN and MoS_2/D-PEN with corresponding roughness values. The scale bar is 500 nm.

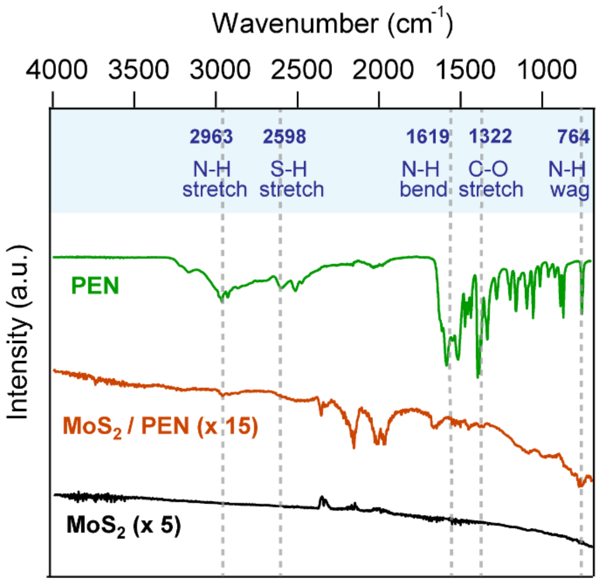

Fig. S3. FTIR spectrum of MoS_2, PEN and MoS_2/ PEN.

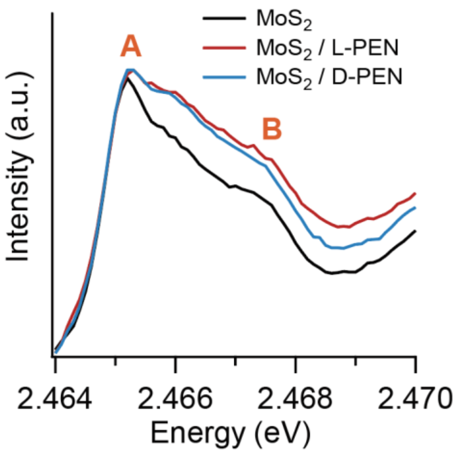

Fig. S4. S K-edge XANES spectra of MoS_2, MoS_2/L-PEN and MoS_2/D-PEN normalized at maximum of band A showing an obvious decrease of peak intensity ratio of band A and B after functionalization with PEN.

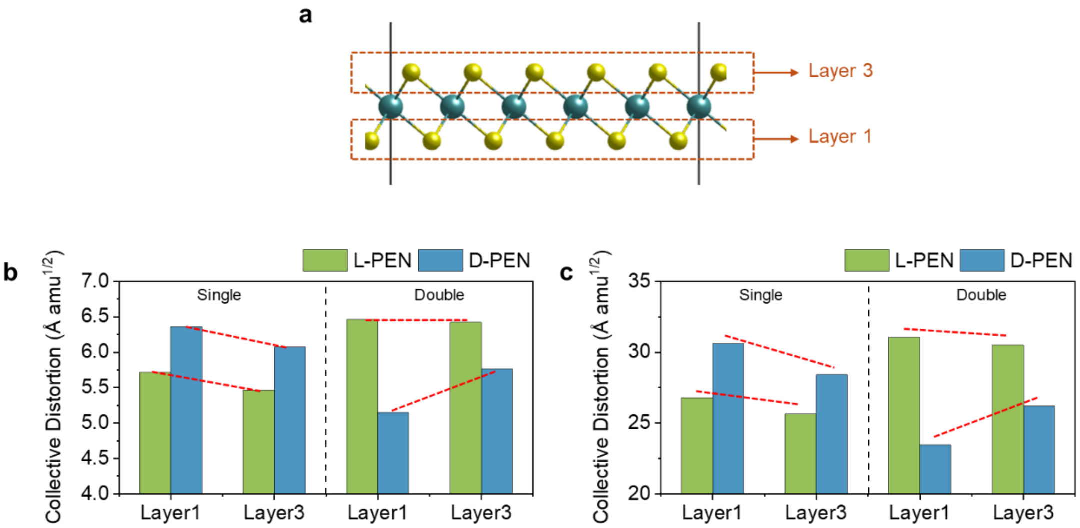

Fig. S5. (a) Illustration of crystal structure for 1T-phase MoS_2 highlighting Layer 1 and Layer 3 with frames. Collective distortion cause by (b) passivated molecules [$\Delta Q = (\sum_i \Delta d_i^2 m_i)^{1/2}$, where d_i and m_i is the displacement magnitude and mass of atom *i*] and (c) by inversion-asymmetric distortions ($\Delta Q_{AS} = \sum_i \Delta d_i {m_i}^{1/2}$).

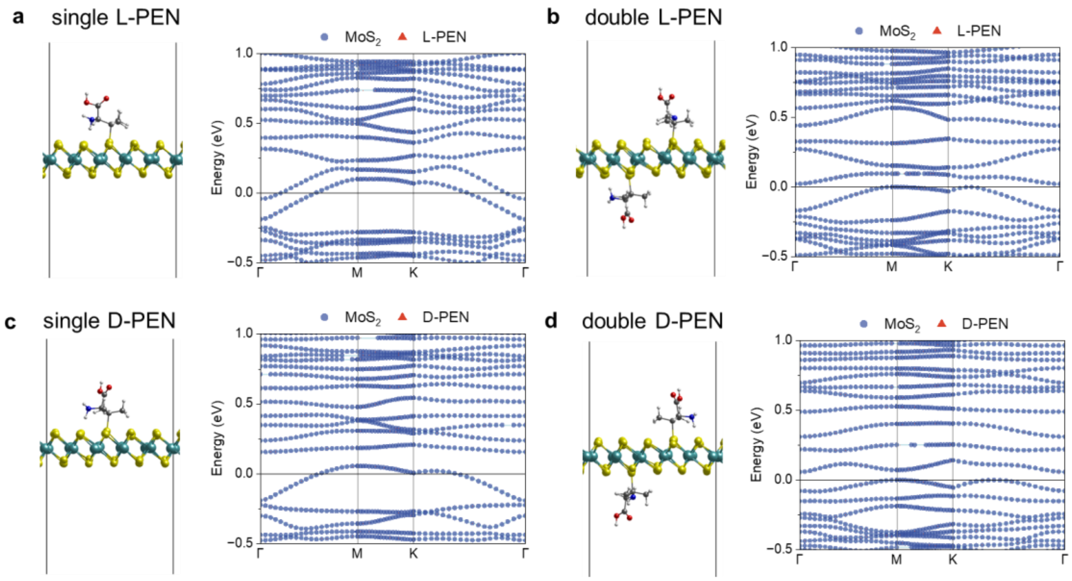

Fig. S6. Optimized crystal structures and electronic bands structures for 1L MoS_2 passivated by (a) single L-PEN, (b) double L-PEN, (c) single D-PEN, and (d) double D-PEN. The DFT calculations were performed at GGA/PBE+vdW level of theory.

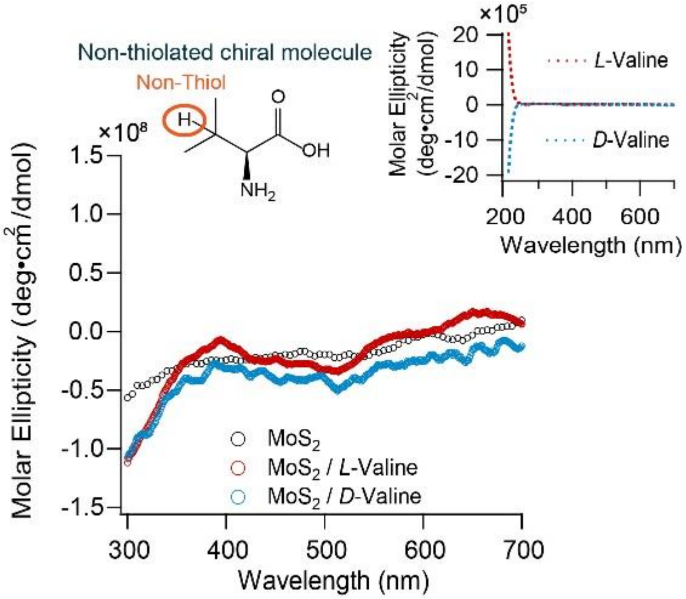

Fig. S7. Molar ellipticity of CVD grown monolayer MoS_2, MoS_2/ L-Valine and MoS_2/ D-Valine showing absence of chirality induction. Valine is not thiolated and therefore does not covalently functionalize with MoS_2.

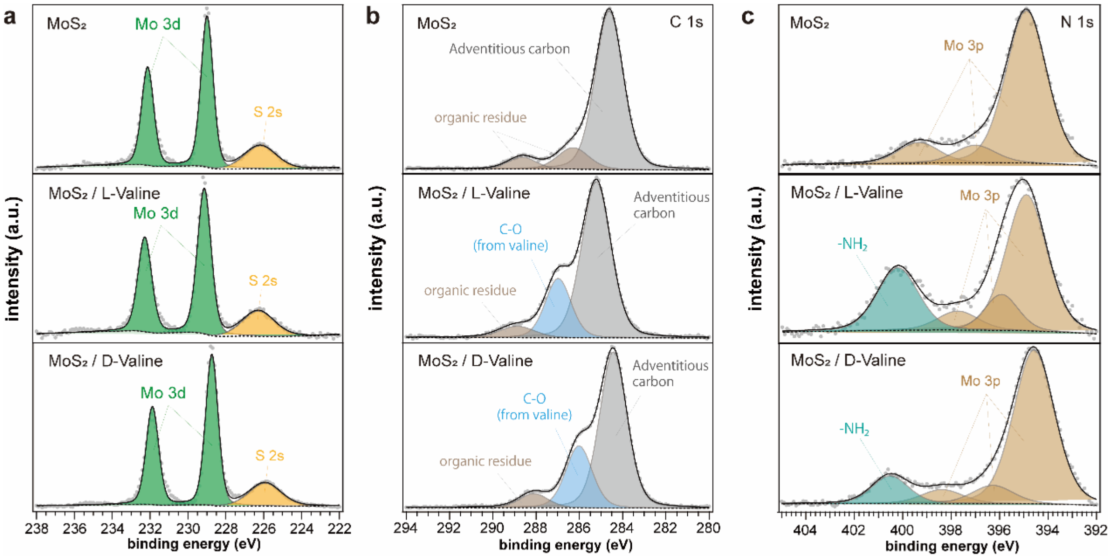

Fig. S8. Core-level (a) Mo 3d, S 2s (b) C 1s and (c) N 1s XPS spectra of MoS_2, MoS_2/L-Valine and MoS_2/D-Valine. The spectra clearly show signals of amino acids (amine group ($-NH_2$) and carbonyl (C-O) group) indicating the presence of molecules on MoS_2 surface.

Excitation	MoS_2	MoS_2/L-PEN	MoS_2/D-PEN
s+	0.865	0.988	0.881
s-	0.761	0.770	0.831

Table S1. E^1_{2g} and A_{1g} Raman peak intensity ratio of MoS_2, MoS_2/L-PEN and MoS_2/D-PEN with right circularly polarized (s+) and left circularly polarized (s-) excitation.

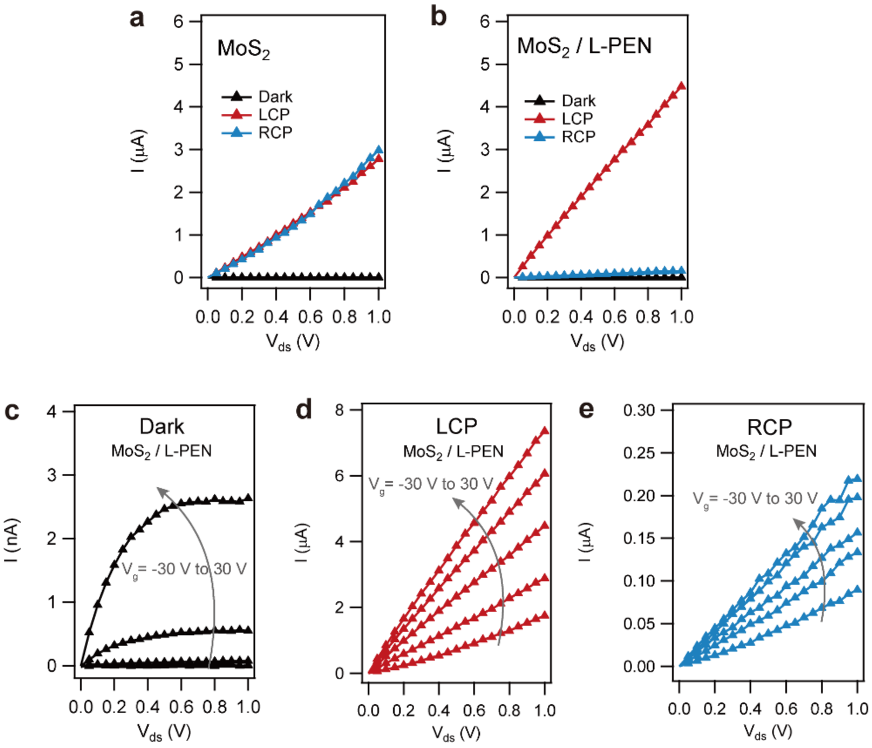

Fig. S9. Output characteristics of MoS_2 and MoS_2/L-PEN phototransistors. (a-b) Output curves of (a) MoS_2 and (b) MoS_2/L-PEN in dark, under LCP and RCP illumination under zero gate bias. (c-e) Gate-dependent output curves of chiral MoS2/L-PEN (c) in dark, (d) under LCP and (e) RCP illumination.

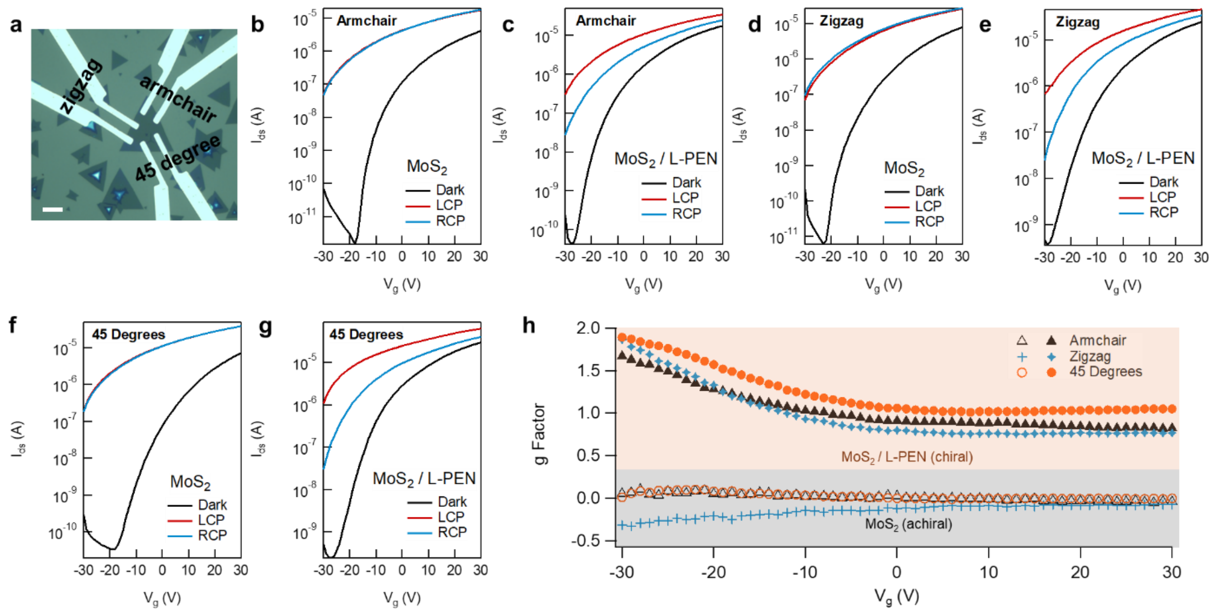

Fig. S10. Effect of orientation of electrodes. (a) Optical image of the studied device. The scale bar is 10 mm. (b)-(g) Photoresponsive transfer curves of (b) (d) (f) MoS_2 and (c) (e) (g) MoS_2/L-PEN with the carrier transport direction along the (b)(c) zigzag, (d)(e) armchair and (f)(g) 45 degrees with respect to the zigzag direction; (h) calculated g factor of MoS_2 and MoS_2/L-PEN with carrier transport direction of armchair, zigzag and 45 degrees direction.

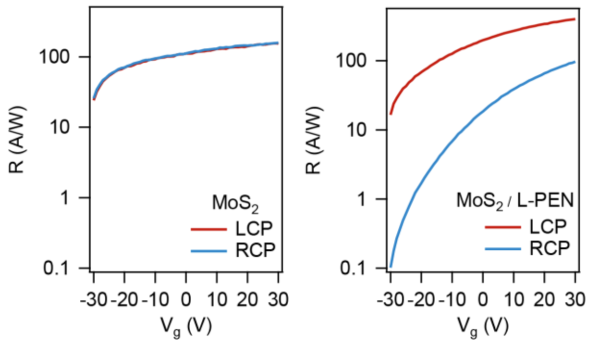

Fig. S11. Gate-dependent photoresponsivity of MoS_2, MoS_2/L-PEN at V_{ds}=1 V.

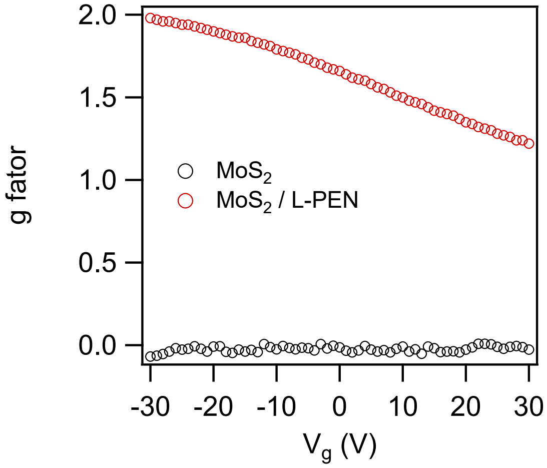

Fig. S12. Gate-dependent g factor of MoS_2 and MoS_2/L-PEN at V_{ds}=1 V.

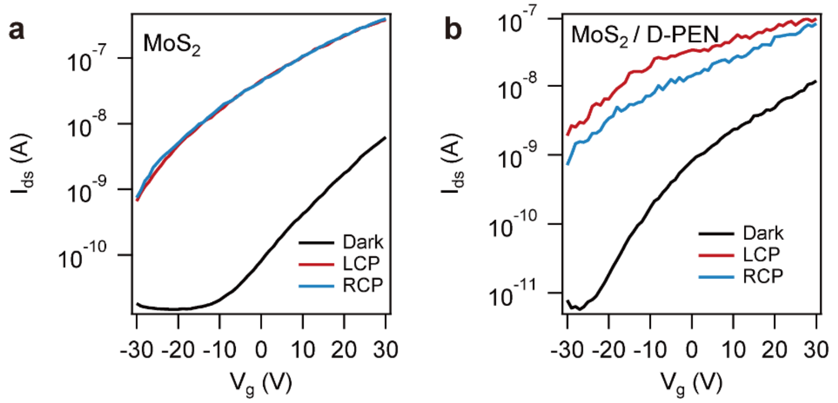

Fig. S13. Gate-dependent photoresponse (a) MoS_2 and (b) MoS_2/ D-PEN illuminated by left and right circularly polarized light (LCP and RCP).

Supporting Text: Power-dependent photoresponse of MoS_2 and MoS_2/L-PEN phototransistors

The power-dependent measurement can be found in Figure S14 below. In a typical device, the pristine phototransistor shows strong power-dependent photocurrent which is similar to previously reported monolayer MoS_2 phototransistors (*Nat. Nanotechnol.* 8, 497–501 (2013)). Photoresponse of left- and right- circularly polarized light illumination at the same light power exhibit same current level. After functionalization with L-PEN, an asymmetric photoresponse appears at various level range where LCP shows much higher photocurrent than RCP. We calculate the power-dependent responsivity (R) of MoS_2 and MoS_2/L-PEN at different gate voltage by $\boldsymbol{R} = \frac{\boldsymbol{I_{ph}}}{\boldsymbol{P \times A}}$ which is plotted in Figure S15. For pristine MoS_2 (Figure S15(a)), At V_g=0V and V_g=+30V, responsivity decreases with incident light power, reaching maximum photoresponsivity up to 10^5 A/W at 1.2 mW/cm^2. At V_g=-30V, peak photoresponsivity of 10^3 A/W is attained at 176.9 mW/cm^2. R of LCP and RCP overlap. After functionalization with L-PEN (Figure S15(b)), only a slight increase of R is seen for all gate voltages (-30 V, 0 V, +30 V), with similar trend as power is increased. LCP exhibits half an order of magnitude larger R than RCP.

To better understand the asymmetric chiral light response in MoS_2/L-PEN, we fit the photocurrent ($\boldsymbol{I_{ph}}$)-light power ($\boldsymbol{P}$) by $\boldsymbol{I_{ph}} \propto \boldsymbol{P^{\gamma}}$ (Figure S15(c)) and obtain a linearity parameter $\boldsymbol{\gamma}$ at different gate voltage under LCP and RCP illumination which is reported in Figure S15(d). $\boldsymbol{\gamma}$ ranges from 0 to 1 where at $\boldsymbol{\gamma}$=1, the photocurrent increases linearly with respect to the incident light power, showing a pure photoconductive effect. At $\boldsymbol{0 < \gamma < 1}$, evolution of photocurrent is non-linear, indicating a complex contribution of photoconductive and photogating effect due to charge trapping at the interface which is a common behavior in 2D materials-based optoelectronics. In the pristine MoS_2 phototransistor, $\boldsymbol{\gamma}$ is identical under LCP and RCP, exhibiting a value between 0.5 and 0.6, which means the photocurrent generation mechanism is combined with photoconductivity and photogating (Nano Lett. 2015, 15, 12, 7853–7858). The small variation of $\boldsymbol{\gamma}$ at different gate voltage reveals that the photocurrent generation mechanism is agnostic to the channel doping level. For MoS_2/L-PEN, $\boldsymbol{\gamma}$ is completely different for LCP and RCP illumination and a strong gate dependence is observed. This means that molecules functionalized on MoS_2 influences the photogating by providing more/less photocarrier traps. Thus, the mechanism of asymmetric chiral photoresponse in chiral MoS_2/L-PEN is possibly a consequence of asymmetric photogating ability when absorbing different amount of circularly polarized photons. Additionally, we calculated specific detectivity (D*) which is defined as $D^* = \frac{I_{ph}\sqrt{A}}{P\sqrt{2qI_{dark}}}$ where q is the elementary charge. As is shown in Figure S16, the highest D* rises to 10^{10} Jones at 1.23 μW/cm^2, comparable to perovskite-based circularly polarized light photodetectors which shows typical values of 10^{11} Jones (Ref [13][17][56] in the main text).

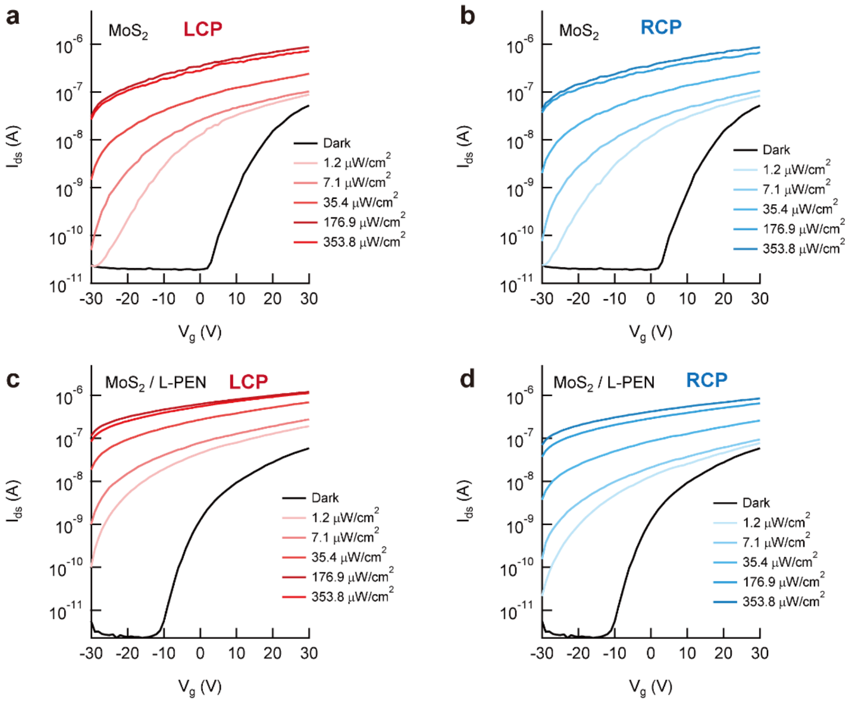

Fig. S14. Power-dependent photoresponse of MoS_2 and MoS_2/L-PEN under circularly polarized light emission. (a)-(b) Power-dependent photoresponse of pristine MoS_2 under (a) LCP and (b) RCP illumination; (c)-(d) Power-dependent photoresponse of pristine MoS_2 under (c) LCP and (d) RCP illumination.

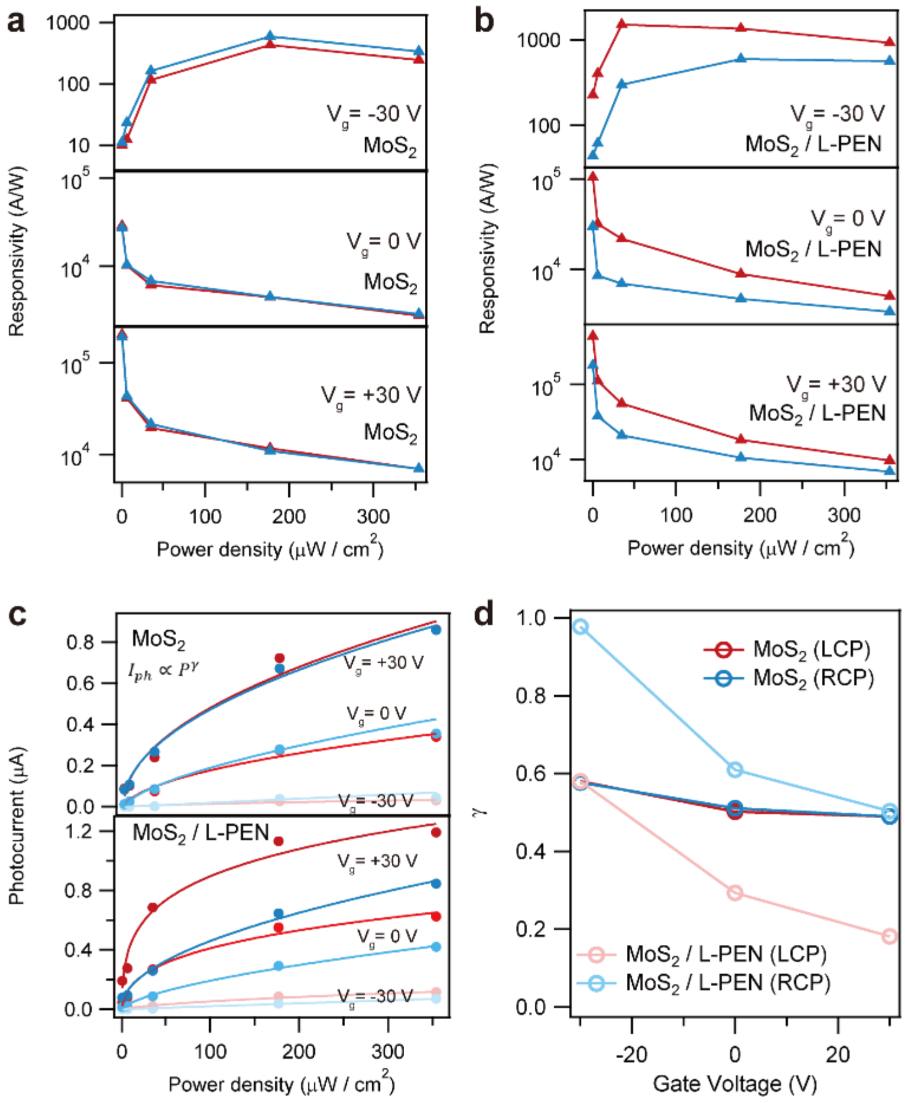

Fig. S15. Calculation and analysis of power-dependent chiral responsivity of MoS_2 and MoS_2/L-PEN. (a) Power-dependent responsivity of MoS_2 at different power density at -30V, 0V and +30V gate voltage. (b) Power-dependent responsivity of MoS_2/L-PEN at different power density at -30V, 0V and +30V gate voltage. (c) Plot of photocurrent versus incident light power. The line is obtained by regression fitting of the equation $\boldsymbol{I_{ph}} \propto \boldsymbol{P^{\gamma}}$; (d) Values of $\boldsymbol{\gamma}$ of MoS_2 and MoS_2/L-PEN transistor under LCP and RCP illumination at different gate voltage obtained from (c).

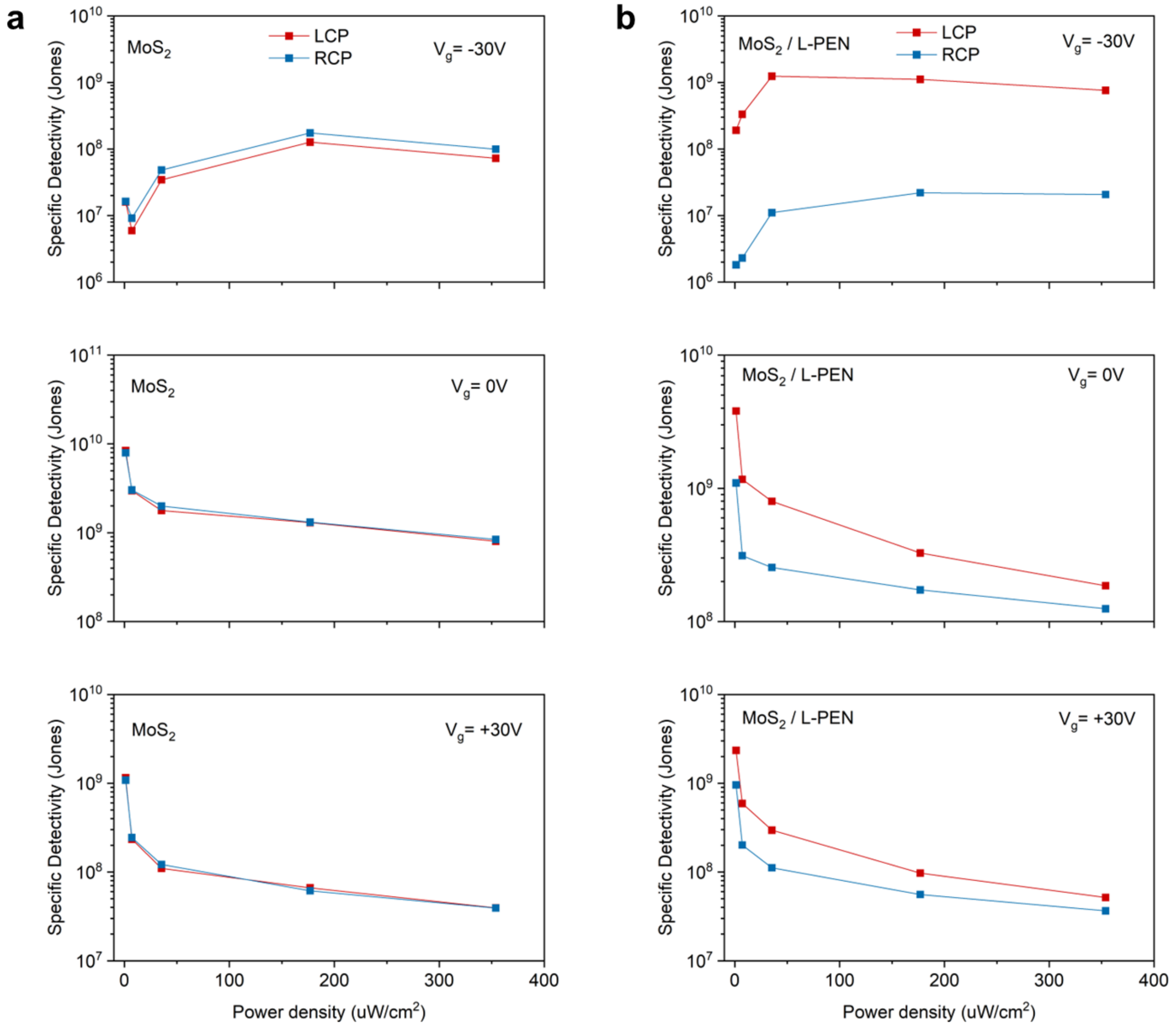

Fig. S16. (a) Power-dependent specific detectivity of MoS_2 at different power density at -30V, 0V and +30V gate voltage. (b) Power-dependent specific detectivity of MoS_2/L-PEN at different power density at -30V, 0V and +30V gate voltage.

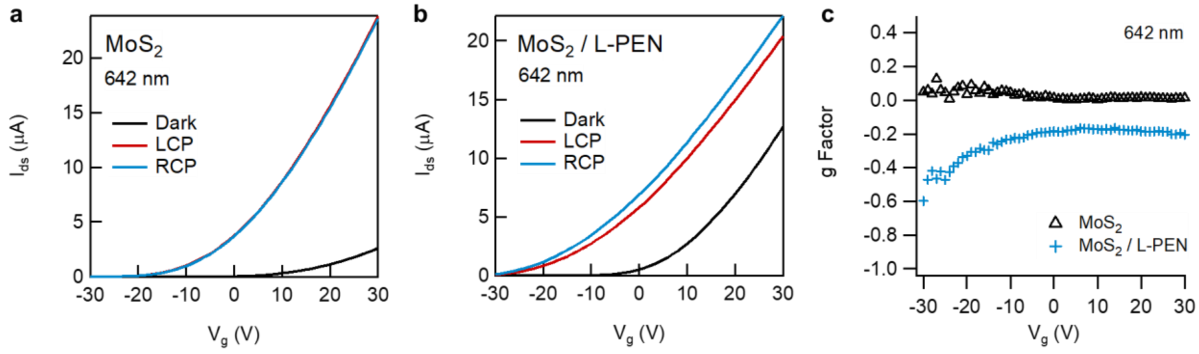

Fig. S17. Photodetection of circularly polarized light at 642 nm. (a) I_{ds}-V_g curves of pristine MoS_2 under 642 nm LCP and RCP illumination. (b) I_{ds}-V_g curves of MoS_2 /L-PEN under 642 nm LCP and RCP illumination. (c) Gate-dependent g factor calculated from (a) and (b). The laser power is 230.2 mW/cm^2.

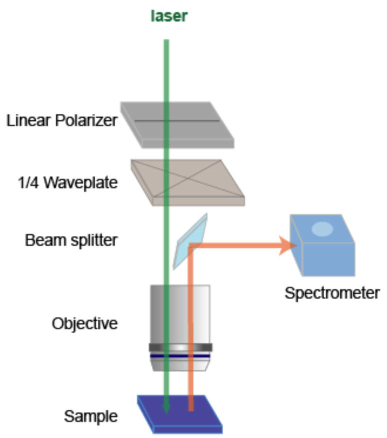

Fig. S18. Optical setup of Raman optical activity measurement.
